# Pevonedistat, a Nedd8-activating enzyme inhibitor, in combination with ibrutinib in patients with relapsed/refractory B-cell non-Hodgkin lymphoma

**DOI:** 10.1038/s41408-022-00763-w

**Published:** 2023-01-11

**Authors:** Pallawi Torka, Swetha Kambhampati Thiruvengadam, Lu Chen, Xiaoguang Wang, Canping Chen, Dan Vuong, Hanjun Qin, Alexandra Muir, Kirsten Orand, Ivana Borja, D. Lynne Smith, Alex F. Herrera, Stephen E. F. Spurgeon, Byung Park, Lionel D. Lewis, Francisco Hernandez-Ilizaliturri, Zheng Xia, Alexey V. Danilov

**Affiliations:** 1 Division of Hematology & Medical Oncology, Roswell Park Cancer Institute, Buffalo, NY USA; 2 City of Hope National Medical Center, Duarte, CA USA; 3 Oregon Health and Science University, Portland, OR USA; 4 Section of Clinical Pharmacology, Dept. of Medicine, The Geisel School of Medicine at Dartmouth and the Dartmouth Cancer Center, Lebanon, NH USA

**Keywords:** Lymphoma, B-cell lymphoma

## Abstract

Pevonedistat (TAK924) is a Nedd8-activating enzyme inhibitor with preclinical activity in non-Hodgkin lymphoma (NHL). This open-label, Phase I, multicenter, investigator-sponsored study enrolled patients with relapsed/refractory (R/R) NHL and chronic lymphocytic leukemia (CLL). The primary objective was safety. Pevonedistat was given intravenously on days 1, 3, 5 of a 21-day cycle for 8 cycles at five dose levels (15 to 50 mg/m^2^); ibrutinib was administered at 420 or 560 mg orally daily continuously. Eighteen patients with NHL were enrolled, including 8 patients with mantle cell lymphoma (MCL) and 4 patients with CLL. One dose-limiting toxicity (mediastinal hemorrhage) occurred at 50 mg/m^2^ of pevonedistat which is the estimated maximum tolerated dose. Bruising and diarrhea were the most common adverse events (56% and 44%). Atrial fibrillation occurred in 3 patients (17%). Grade ≥3 toxicities included arthralgia, atrial fibrillation, bone pain, diarrhea, hypertension, and mediastinal hemorrhage (one patient each). The overall response rate (ORR) was 65% (100% ORR in MCL). Pevonedistat disposition was not modified by ibrutinib. scRNA-Seq analysis showed that pevonedistat downregulated NFκB signaling in malignant B-cells in vivo. Thus, pevonedistat combined with ibrutinib demonstrated safety and promising early efficacy in NHL and CLL. NAE inhibition downregulated NFκB signaling in vivo.

## Introduction

Non-Hodgkin lymphoma (NHL) is a heterogeneous group of lymphoid neoplasms accounting for 4.3% of new cancer cases diagnosed yearly in the United States [1]. Many patients with B-cell malignancies cannot be cured. In patients with diffuse large B-cell lymphoma (DLBCL), 3-year progression-free survival (PFS) ranges between 40% and 74% depending on the cell of origin [2]. Meanwhile, while most patients with mantle cell lymphoma (MCL) and chronic lymphocytic leukemia (CLL) achieve remissions following initial therapy, relapses are inevitable. Thus, novel approaches are needed to address this unmet need.

Pevonedistat (TAK924, MLN4924) is a first-in-class, small molecule inhibitor of NEDD8-activating enzyme (NAE) that selectively prevents activation of Cullin-RING ligases (CRLs) and thereby diminishes proteasomal degradation of CRL substrates [3]. NAE inhibition demonstrated preclinical efficacy in multiple cancer models, including DLBCL [3–5]. We have shown that treatment with pevonedistat abrogated the pro-survival effect of the lymph node-mimicking conditions via downmodulation of NFκB signaling and upregulation of the pro-apoptotic BCL2 family proteins BAX and NOXA in resting CLL cells [6, 7]. In the proliferative CLL cell fraction, NAE inhibition upregulated the replication-licensing factor CDT1, leading to DNA damage and cell cycle arrest [8]. In DLBCL, treatment with pevonedistat led to diminished NFκB signaling and downregulated expression of BCL2/XL but activated BAK [4, 5]. Finally, we have confirmed the pre-clinical efficacy of NAE inhibition in MCL models via a similar mechanism, where it also enhanced the antitumor activity of chemotherapeutic agents as well as rituximab [9]. While pevonedistat was well tolerated in an early phase clinical trial in patients with NHL and multiple myeloma, it had limited clinical efficacy as a single agent [10].

Chronic active B-cell receptor (BCR) signaling pathway has been implicated in the pathogenesis of B-cell neoplasia [11]. Assimilation of the inhibitors of BCR-associated kinases such as Bruton tyrosine kinase (BTK) inhibitors and phosphoinotiside-3 kinase δ (PI3Kδ) inhibitors into clinical practice has shifted the treatment paradigm in lymphoid malignancies. The BTK inhibitor ibrutinib was associated with an overall response rate (ORR) of 68% in a pivotal Phase II trial in patients with R/R MCL [12] and ORR of 91% in a pivotal phase III trial in R/R CLL [13]. However, ibrutinib induces complete responses (CR) in only a fraction of patients [14]. Furthermore, patients with R/R MCL or CLL who develop ibrutinib resistance are high-risk with poor survival outcomes (median OS of 9.3 months in CLL [15] and 5.6 months in MCL [16]). Lastly, ibrutinib demonstrates low efficacy in histologies such as DLBCL and follicular lymphoma (FL).

We have demonstrated that NAE inhibition sensitized primary CLL and MCL cells to BCR-signaling inhibitors, including ibrutinib and idelalisib, in microenvironment-mimicking conditions [6]. Furthermore, our group established that the combination of pevonedistat and ibrutinib was synergistic in DLBCL cell lines in vitro, and led to prolonged survival of DLBCL xenograft mice compared with either drug alone, without significant added toxicity [5]. Based on this pre-clinical rationale, we hypothesized that pevonedistat plus ibrutinib will be well tolerated in patients with R/R NHL and designed an open label, dose escalation Phase I trial to investigate the safety, tolerability, and preliminary efficacy of this novel combination in patients with NHL/CLL.

## Methods

### Study design and participants

This was an investigator-sponsored, open-label, single arm, multicenter, dose escalation phase I study conducted at City of Hope National Medical Center (Duarte, CA), Roswell Park Comprehensive Cancer Center (Buffalo, NY) and Oregon Health and Science University (Portland, OR). The protocol was approved by each clinical site’s institutional review board and the study was conducted in accordance with Good Clinical Practice Guidelines and the Declaration of Helsinki. All patients provided written informed consent prior to the performance of any study procedures. The trial was registered on clinicaltrials.gov (NCT03479268).

The study followed a modified toxicity probability interval (mTPI) design to determine the maximum tolerated dose (MTD) and the recommended Phase 2 dose (RP2D) of pevonedistat and to evaluate the safety and preliminary efficacy of its combination with ibrutinib.

Eligible patients were aged ≥18 with histologically confirmed R/R B-cell NHL/CLL. Patients had received ≥1 prior therapy and had either documented disease progression or no response to the most recent treatment regimen. Patients must have had measurable disease, met criteria for treatment, had ECOG performance status ≤2 and good organ function as follows: total bilirubin ≤institutional upper limit of normal (ULN), aspartate/alanine aminotransferases (AST/ALT) ≤ 3XULN; estimated creatinine clearance [Cockroft-Gault] ≥30 mL/min; platelets ≥50,000/mm^3^ independent of transfusion support with no active bleeding, absolute neutrophil count ≥1000/mm^3^, unless due to disease involvement in the bone marrow.

### Study treatment

Treatment schema is shown in Supplementary Table 1. The five planned dose levels (DL) of pevonedistat were 15, 20, 25, 37.5 and 50 mg/m^2^ (DL“-1” at 10 mg/m^2^ was also defined, if needed). A dose of 50 mg/m^2^ was the highest planned dose of pevonedistat due to earlier reports of toxicities at higher doses in patients with acute myeloid leukemia [17]. Dose escalation occurred per the modified toxicity probability interval (mTPI) design [18]. Pevonedistat was administered intravenously on days 1, 3, and 5 of each 21-day cycle for up to 8 cycles. Beginning with cycle 1 day 2, ibrutinib was given at a fixed oral daily dose of 420 mg (560 mg for MCL) for up to 18 cycles. Continuation of ibrutinib beyond 18 cycles was left at Investigators’ discretion. A time-limited strategy was pursued in an effort to minimize long-term toxicities of BTK inhibition.

Grading of toxicities was according to NCI CTCAE v.4.03, except hematologic toxicities in patients with CLL which were graded per IWCLL 2008 criteria [19]. Dose limiting toxicities (DLTs) were assessed during the first cycle of treatment. DLTs were defined as grade 4 neutropenia lasting >5 days in any subject with ANC > 1000/mm^3^ before beginning therapy; febrile neutropenia; grade 4 thrombocytopenia or grade 3 thrombocytopenia with bleeding or any requirement for platelet transfusion unexplained by underlying disease; grade 4 anemia unexplained by underlying disease; any grade ≥3 non-hematologic toxicity (excluding grade 3 nausea, vomiting, diarrhea or infusion-related toxicity reversible within 72 h; grade 3 fatigue which lasted <7 days; asymptomatic grade 3 laboratory abnormalities reversible to ≤grade 2 within 72 h; grade 3 tumor lysis syndrome of ≤7-day duration, hyponatremia); other grade ≥2 non-hematologic toxicities that, in the opinion of the investigator, required a dose reduction or discontinuation of therapy with pevonedistat; grade ≥2 elevation of the PT/aPTT with clinically significant bleeding.

### Pevonedistat plasma concentration measurements and pharmacokinetic data analysis

For PK analysis, plasma samples were collected on C1D1 [pre-infusion, end of infusion (immediately prior to switching off the infusion), 1 and 3 h after completion of infusion]; C1D2 before administration of ibrutinib, C1D3 (pre-infusion, end of infusion, and 1, 3 and 7 h after completion of infusion), C1D4 before administration of ibrutinib, C1D5 (pre-infusion, end of infusion. In addition, optional collections occurred 7 h after completion of infusion on C1D1 and 1, 3, and 7 h after completion of the pevonedistat infusion on C1D5.

Pevonedistat plasma concentrations were measured in samples using a GLP-validated liquid chromatography/tandem mass spectrometry (LC-MS/MS) assay. Plasma pevonedistat concentration vs time profiles were initially inspected on a semi-logarithmic plot of concentration vs time. Pharmacokinetic analysis was then performed on the plasma pevonedistat concentration over time data using non-compartmental modeling for a constant intravenous infusion in PC-WinNonlin, model 202. For individual patient data on different days, the pevonedistat elimination rate constant (λ) was estimated by linear regression of the terminal three to six log plasma pevonedistat concentration-time data points. The pevonedistat half-life was estimated from 0.693/λ; clearance (CL) was estimated from the pevonedistat dose divided by the area under the concentration time curve from time 0 to infinity [AUC(0-inf)] and the apparent volume of distribution (Vd) was estimated from CL/λ. The maximum pevonedistat concentrations (C_max_) and the time to maximum plasma pevonedistat concentration (T_max_) were the observed values.

### Statistical analysis

Dose finding adopted a mTPI design with a target DLT rate of 15% with a proper dosing interval of 10–25%. At least 6 patients were to be treated at the recommended Phase 2 dose (RP2D). Analyses of safety were performed for all patients who had received at least one dose of pevonedistat. For DLT evaluations, patients needed to receive 3 doses of pevonedistat and 14+ doses of ibrutinib in cycle 1 or had DLT in cycle 1 to be evaluable.

All adverse events (AE) were tabulated using descriptive statistics. Response was evaluated per Lugano criteria in patients with NHL and per IWCLL 2008 criteria in CLL [20, 21]. Event-free survival (EFS) was defined as the time from first study treatment to the date of objective signs of disease recurrence, subsequent anticancer therapy, or death, whichever occurred first. The Kaplan–Meier product limit method was used to estimate EFS rates at clinically relevant time points; 95% CI was estimated based on Greenwood variance estimator and log-log transformation.

Summary pevonedistat pharmacokinetic parameters are presented as median (range) because of the small cohort sizes. Comparisons of PK parameters between days 1 and 3 of study treatment used the Wilcoxon matched-pairs signed rank test (non adjusted for multiple comparisons). For comparisons of C_max_ between days 1, 3, and 5 the Friedman non-parametric RMANOVA test was used.

## Results

### Patient characteristics

Of the 18 patients enrolled, 8 had MCL, 4 had DLBCL, 4 had CLL (including 1 B-PLL) and one patient each had marginal zone lymphoma and FL (Table 1). Median age was 71 years (range, 51–90) and 72% (13/18) were men. ECOG performance status was ≤1 in 94% of patients. Patients had received a median of 1 prior line of therapy (range, 1–3). List of prior therapies by lymphoma subtype are shown in Supplementary Table 2. Three patients had undergone prior autologous stem cell transplant, all of whom had MCL and underwent stem cell transplant as consolidative therapy in first remission (one achieved CR and two achieved PR before progressing).

**Table 1:** Baseline patient characteristics.

Baseline demographics	All patients (*N* = 18)
Age, median (range), *y*	71.0 (50.7–90.1)
Sex, *n* (%)	
Male	13 (72.2)
Ethnicity	
Non-Hispanic	18 (100)
Race	
White	17 (94.4)
Asian	1 (5.6)
ECOG performance status, *n* (%)	
0	13 (72.2)
1	4 (22.2)
2	1 (5.6)
Diagnosis, *n* (%)	
Mantle cell lymphoma (MCL)	8 (44.4)
Diffuse large B-cell lymphoma (DLBCL)	4 (22.2)
Chronic lymphocytic leukemia (CLL) and B-PLL	4 (22.2)
Follicular lymphoma (FL)	1 (5.6)
Marginal zone lymphoma (MZL)	1 (5.6)
Median no. of prior lines of therapy (range)	1 (1–3)
Prior autologous stem cell transplant, *n* (%)	3 (16.7)

Three patients each were treated on dose levels 1–4 and six patients were treated at dose level 5. All patients are off treatment. Median duration of treatment was 8.3 months (range, 1.0–13.2 months). Twelve patients received all pevonedistat doses for 8 cycles; the other 6 patients terminated after a median of 3.5 (range 1–6) cycles. One patient received 75% of pevonedistat doses for the pevonedistat cycles due to hospitalization and atrial fibrillation. Two patients had pevonedistat dose reduction and four patients had ibrutinib dose reduction during treatment, all due to adverse events. Progressive disease was the most common reason of early discontinuation (*n* = 9; 50%), other reasons for treatment termination included treatment completed (*n* = 5, 27.8%), adverse events (*n* = 3; 16.7%) and death due to unrelated causes while on treatment (*n* = 1, 5.6%).

### Safety

No DLTs were observed during the first four dose levels of pevonedistat (15–37.5 mg/m^2^). One DLT, mediastinal hemorrhage with cardiac tamponade, occurred at dose level 5 (50 mg/m^2^) in a patient with CLL after 2 weeks of combination therapy, resulting in treatment discontinuation. The patient was subsequently treated with duvelisib. Therefore, the MTD and the recommended phase 2 dose (RP2D) of pevonedistat was determined as 50 mg/m^2^. The most common treatment-related adverse events (AEs, any grade) were bruising and diarrhea (56% and 44%; Table 2). Three patients (16.6%) developed atrial fibrillation on study, and two discontinued while one continued on treatment and completed protocol therapy. There was one case each (5.6%) of grade ≥3 AEs, including arthralgia, bone pain, diarrhea, hypertension, as well as the previously noted atrial fibrillation and mediastinal hemorrhage/cardiac tamponade. All of these grade ≥3 AEs occurred during the first eight cycles when patients were on both pevonedistat and ibrutinib with the exception of hypertension which occurred after discontinuation of pevonedistat but spontaneously resolved without requiring any dose reduction or discontinuation of ibrutinib.

**Table 2:** Safety profile: treatment-related adverse events.

Adverse event	*N* (%)*
All Grades	
Bruising	10 (56%)
Diarrhea	8 (44%)
Musculoskeletal and connective tissue disorder - Other, specify	6 (33%)
Fatigue	6 (33%)
Platelet count decreased	4 (22%)
Anemia	4 (22%)
Nausea	3 (17%)
Atrial fibrillation	3 (17%)
Neutrophil count decreased	2 (11%)
Bone pain	2 (11%)
Headache	2 (11%)
Gastrointestinal disorders - Other, specify	2 (11%)
Epistaxis	2 (11%)
Noncardiac chest pain	2 (11%)
Rash maculo-papular	2 (11%)
Peripheral sensory neuropathy	2 (11%)
Muscle cramp	2 (11%)
Alkaline phosphatase increased	2 (11%)
Hypertension	2 (11%)
Grade 3–4	
Diarrhea	1 (5.5)
Bone pain	1 (5.5)
Arthralgia	1 (5.5)
Atrial fibrillation	1 (5.5)
Mediastinal hemorrhage	1 (5.5)
Cardiac tamponade	1 (5.5)
Hypertension	1 (5.5)

*Any-grade AEs reported in ≥ 10% of patients (2 + ) in the total population; All treatment related grade 3–4 AEs reported.

### Efficacy

**Fig. 1:**
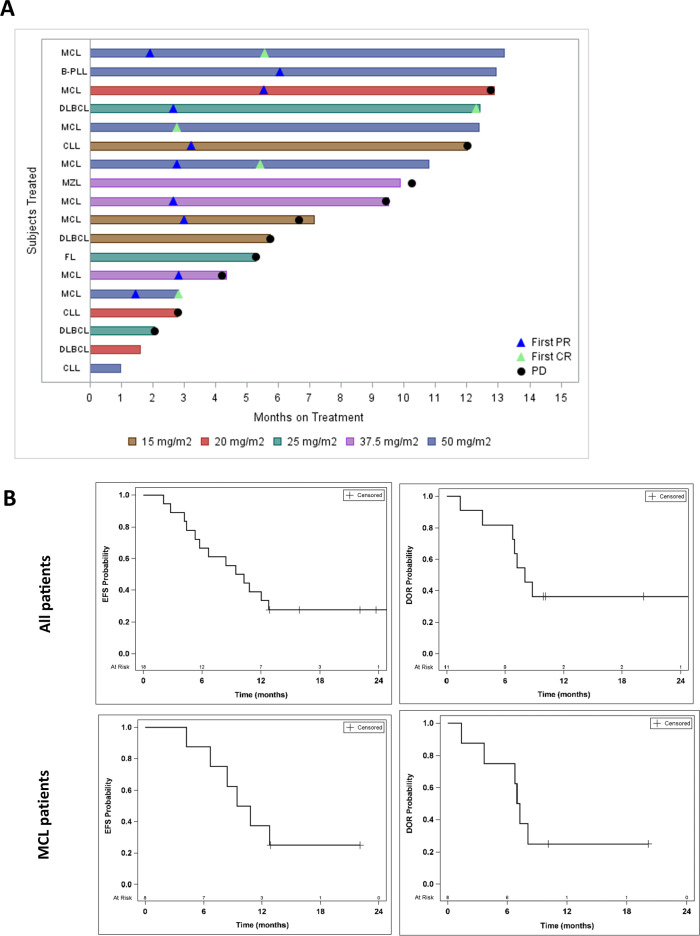
Clinical efficacy of pevonedistat plus ibrutinib in NHL. **A** Swimmer’s plot of patients and responses on treatment. **B** Kaplan–Meier EFS and DOR curves for all participants (*n* = 18) and for MCL only (*n* = 8).

The ORR was 65% for all patients (*n* = 17 response evaluable) with 29% of patients achieving CR and 35% of patients achieving PR. The swimmer’s plot of all patients and their responses on treatment by subtype and dose is shown in Fig. 1A. Overall, 13 patients had experienced EFS failures (disease progression in 11 patients, start of new therapy in 1 patient, and death in 1 patient). At a median follow-up of 22.1 months (range 12.9–33.1), the median EFS was 9.8 months (95% CI: 5.3–12.8). Among responders, median duration of response (DOR) was 8.0 months (95% CI: 3.7 – NA; Table 3 and Fig. 1B). Four patients remain in follow-up after completion of 18 cycles of therapy, including one patient with heavily pretreated DLBCL. The characteristics of these patients who completed study treatment are shown in Supplementary Table 3.

**Table 3:** Efficacy data.

	All patients *N* = 18	MCL *N* = 8	CLL/B-PLL *N* = 4
Best Response, *N* (%)^a^			
ORR (CR + PR)	11 (65)	8 (100)	2 (50)
CR	5 (29)	4 (50)	0 (0)
PR	6 (35)	4 (50)	2 (50)
SD	4 (24)		1 (25)
PD	2 (12)		1 (25)
Median follow-up (months)^b^, range	22.1 (12.9–33.1)	12.9, 22.1	15.9
Median EFS, months (95% CI)	9.8 (5.3–12.8)	10.1 (4.2–NA)	8.2 (2.8–NA)
1-year EFS (95% CI)	38.9% (17.5–60.0%)	37.5% (8.7–67.4%)	50% (5.8–84.5%)
Median DOR, months^c^ (95% CI)	8.0 (3.7–NA)	7.1 (1.4–NA)	8.8, 9.9+

^a^One DLBCL subject excluded (received 1 cycle and no response assessment).

^b^Among subjects without EFS events (*n* = 5, 2, and 1 for all, MCL, and CLL/B-PLL respectively).

^c^Among responders (*n* = 11, 8, and 2 for all, MCL, and CLL/B-PLL, respectively).

Eight patients with MCL enrolled on study, four patients had complex karyotype (including two with concurrent aberrant *TP53*) and one additional patient had blastoid variant, thus representing high-risk disease. Despite this, ORR was 100% and 50% of MCL patients achieved CR. Median EFS was 10.1 months and median DOR was 7.1 months.

There were four patients with DLBCL in the study, one of whom achieved a CR. The characteristics of these patients are shown in Supplementary Table 4.

Finally, among patients with CLL (*n* = 4; Table 3), ORR was 50% (all PR).

### Pharmacokinetics and pharmacodynamics

**Fig. 2:**
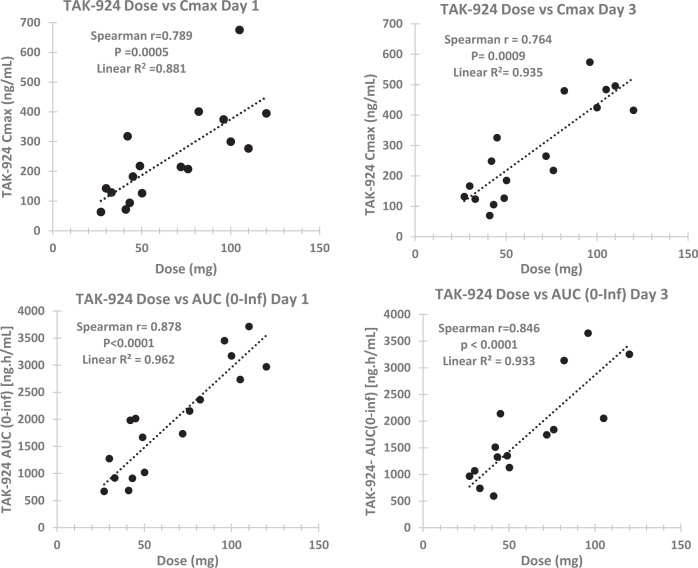
Pharmacokinetics of pevonedistat. Pevonedistat C_max_ and AUC vs dose on days 1 and 3.

The primary pevonedistat pharmacokinetic parameters in the dose cohorts studied on day 1 and day 3 are summarized in Supplementary Table 5. Comprehensive pharmacokinetic parameters for pevonedistat on day 5 were limited to C_max_ due to the short duration of the sampling post dosing (data not shown). Between days 1 and 3 there was no significant change in pevonedistat T½ (unadjusted Wilcoxon *p* = 0.12), AUC (unadjusted Wilcoxon *p* = 0.93), apparent volume of distribution-Vd (unadjusted Wilcoxons *p* = 0.89) or clearance (unadjusted Wilcoxon *p* = 0.23). Similarly, for pevonedistat C_max_ there was no significant change between days 1, 3 and 5 (Friedman non parametric RMANOVA *p* = 0.19). Pevonedistat C_max_ and AUC increased linearly with dose over the range of doses studied as shown in Fig. 2.

### Pevonedistat downregulates NFκB signaling in malignant B cells

We evaluated the tumor-intrinsic effects of pevonedistat. Although we acknowledge the critical role of the lymph node niche on NHL cells, it was not feasible to procure serial specimens from that compartment. Thus, peripheral blood mononuclear cells (PBMC) were collected from patients at baseline and 3 and 24 h after pevonedistat infusion (prior to ibrutinib initiation which occurred on C1D2). Paired samples from 4 patients were submitted for scRNA-Seq (3 with MCL, one with DLBCL; Supplementary Table 6). We concentrated further on the effect of pevonedistat on malignant B cells. Samples PV7 and PV23 had no cell clusters identified as B-cells and were not analyzed further.

**Fig. 3:**
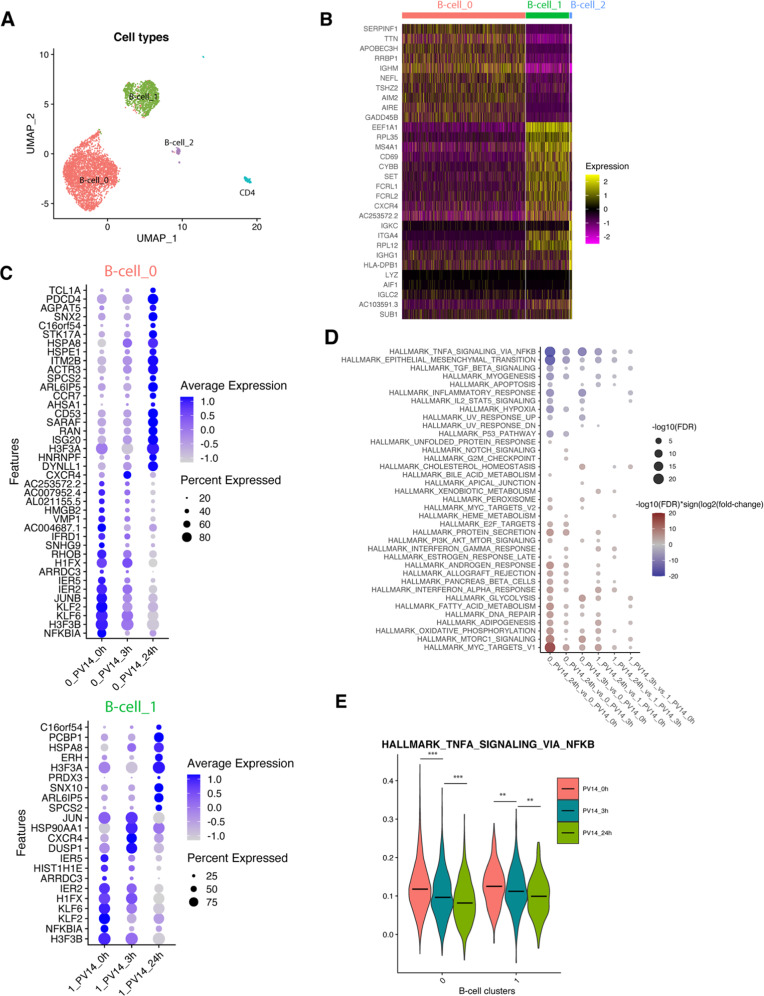
scRNA-seq profiling of immune cells in MCL. **A** UMAP (uniform manifold approximation and projection) visualization of all immune cells colored by immune cell type. **B** Heat map showing scaled expression of the top 10-fold change ranked marker genes of each B cell cluster. **C** Dot plots show the expression levels of signature genes of each condition within B-cell_0 and B-cell_1 clusters. The size of the dot encodes the percentage of cells expressing each gene and the color encodes the average expression level. **D** Dot plots show the statistical differences of pathway enrichment scores between two times in B-cell_0 and B-cell_1 clusters, respectively. The size of the dot encodes the FDR between two time points, while the color encodes the directions of changes by multiplying the FDR values with the sign of the log_2_ fold changes. Red indicates a higher enrichment score in the first condition of the comparison indicated at x axis. **E** Violin plots show the comparisons of enrichment scores of the HALLMARK_TNFA_SIGNALING_VIA_NFKB pathway of cells across three time points within B-cell_0 and B-cell_1 clusters. The Wilcoxon signed-rank test (two-sided) with FDR are shown. **FDR < 0.01, ***FDR < 0.001.

Meanwhile, sample PV14 revealed predominance of B-cells. After quality control, we obtained 4,633 cells (median number of genes per cell – 2138; range, 501–4687). In total, we identified 4 clusters across 2 distinct lineages: B cells and CD4^+^ T cells (Fig. 3A). No natural killer or myeloid cells were detected in this sample. There was no clustering by collection timepoints (Supplementary Fig. S1A), suggesting negligible batch effects because of our multiplexing strategy. Three distinct B-cell clusters were identified, exhibiting distinct gene expression patterns (Supplementary Table 7), with the top 10 most differentially expressed genes of each cluster visualized in the heatmap (adjusted *p* < 0.05; Fig. 3B). InferCNV analysis revealed chr6 deletion for both clusters 0 and 1. Furthermore, we detected chr9 deletion specifically in B-cell cluster 0, while *SOX11* mRNA transcript was overexpressed in B-cell cluster 1 (Supplementary Fig. S1B, C). Thus, our analysis identified two types of malignant B-cells, both carrying deletion of chr6; one clone had chr9 deletion and was SOX11-negative, while another was SOX11-positive, suggesting a heterogeneity of malignant cells in this MCL patient [22]. Since the third cluster only had 68 cells, we focused on the two large B-cell clusters in the follow analysis.

We next evaluated genes deregulated by pevonedistat treatment in the two B-cell clusters (Fig. 3C). B-cell cluster 0 had higher number of significantly differentially expressed genes compared with cluster 1. We found downregulation of *NFKBIA* mRNA following pevonedistat treatment in both clusters, likely related to a negative feedback loop given that IκBα is a known CRL substrate. Another downregulated gene KLF6 was also previously identified as a CRL substrate [23]. Interestingly, *CXCR4* was upregulated by 3 h and reduced by 24 h in both clusters (Fig. 3C). The deregulated pathways confirmed on-target effect of pevonedistat. Consistent with our pre-clinical data [6], NFκB signaling-related genes were included in the top pathways downregulated following pevonedistat treatment, i.e., “TNFα signaling via NFκB”, inflammatory response, apoptosis and IL2-STAT5 signaling (Fig. 3D, E). Other downregulated pathways were also consistent with known CRL substrates and oncogenes, such as hypoxia-related pathways (HIF-1α), “P53”, “UV response” and “G2M checkpoint” (*JUN, CDKN1A, CCND3, NFKBIA, DUSP1* were example genes represented in these pathways).

These results were broadly confirmed in sample PV24 with 1263 cells obtained. In this sample, 5 distinct clusters were identified, including CD4^+^ T-cells, NK-cells, CD16^+^ and CD16^-^ Monocytes and B cells (Supplementary Fig. S2A, B). 139 out of 1263 cells were identified as B-cells (Supplementary Fig. S2C). Likely due to low cell numbers, only one gene was identified as the signature gene of B-cells upregulated at 24 h at a statistically significant level (vs. the other two timepoints; Supplementary Fig. S2D), and no signature genes were found at 0 and 3 h. Nevertheless, consistent with results obtained in sample PV14, we observed downregulated “TNFα signaling via NFκB” following pevonedistat exposure in this sample as well, particularly at the 24-h timepoint (Supplementary Fig. S2E).

Thus, using primary samples from patients treated with pevonedistat we observed rapid deregulation of multiple genes, in particular downregulation of NFκB signaling in the malignant B cells.

## Discussion

In this report, we for the first time investigated the combination of the NAE inhibitor pevonedistat and the BTK inhibitor ibrutinib in patients with NHL. This combination was found to be safe with the majority of reported AEs being typical of ibrutinib therapy (atrial fibrillation, bruising, diarrhea, arthralgias). Only one DLT (mediastinal hemorrhage, at 50 mg/m^2^, which is the estimated MTD) was observed, and along with the two cases of atrial fibrillation, contributed to three patients discontinuing study therapy due to AEs. Although this study was small with a relatively short follow-up, the frequency of AEs appeared not in excess of what is typically expected with ibrutinib therapy [24]. Most common AEs of diarrhea and bruising occurred with a frequency of 56% and 44% in our study, similar to that reported in a long-term follow-up study of single agent ibrutinib in R/R mantle cell lymphoma in which diarrhea and bleeding was observed in over 50% of patients [25]. Atrial fibrillation occurred in 16.7% of patients. This is significant, but likely comparable to a recent population-based cohort study where 1-year incidence of atrial fibrillation was ~13% among patients with CLL treated with ibrutinib, possibly attributable to Src kinase inhibition [26, 27]. In this study, the 1-year frequency of hospital-diagnosed bleeding was ~5%. Overall, data suggest that NAE inhibition did not augment BTK inhibitor-related toxicities or result in new prohibitive AEs.

Preliminary efficacy was noted all NHL subtypes with an ORR of 65% and CR rate of 29%. The combination of ibrutinib and pevonedistat was particularly active in patients with MCL with an ORR of 100% and CR rate of 50% though DOR was short at 7.1 months. Ibrutinib as a single agent demonstrated a lower ORR of 68% and CR rate of 21% but longer DOR of 17.5 months in patients with R/R MCL leading to its approval in this setting [12]. Many subsequent studies have sought to improve the efficacy of ibrutinib by employing combination regimens. Ibrutinib has demonstrated activity in R/R MCL in doublets with agents such as venetoclax, palbociclib, ublituximab and bortezomib as well as in triplets with bendamustine-rituximab, lenalidomide-rituximab, and obinutuzumab-venetoclax [28–34]. This study provides preliminary evidence that ibrutinib in combination with pevonedistat compares favorably to these other combinations, including palbociclib-ibrutinib (ORR 67%; CR 37%), venetoclax-ibrutinib (ORR 71%; CR 60%) or lenalidomide-rituximab-ibrutinib (ORR 71%; CR 60%) [28, 31, 34] though the responses do not appear to be durable.

Pevonedistat is mainly metabolized by CYP3A and to a lesser extent CYP1A2 and is also an ABCB1/P-gp drug transporter substrate. The pevonedistat pharmacokinetics parameters observed in this study in CLL patients on day 1 are consistent with published data from pevonedistat monotherapy studies in patients with solid tumors, over the dose range 15–50 mg/m^2^ and in patients with acute myeloid leukemia either as monotherapy or in combination with azacytidine [17, 35–38]. Ibrutinib is a weak time-dependent inducer of CYP3A and a weak competitive inhibitor of CYP2B6 function in vitro, but these effects are not considered to be clinically significant at the recommended ibrutinib doses [37]. Consistent with this, we did not observe significant changes in the pevonedistat half-life, C_max_ or AUC after two days of ibrutinib therapy and there was no significant change in pevonedistat C_max_ after 4 days of ibrutinib therapy. These data are suggestive of there being no drug-drug interaction between ibrutinib and pevonedistat. Interestingly a drug-drug interaction study with fluconazole/itraconazole (strong CYP3A and ABCB1 inhibitors) showed there was no clinically significant changes in pevonedistat pharmacokinetic disposition, specifically AUC [37]. In our study, we only obtained pharmacokinetic data over 4 days of combination therapy and rich pharmacokinetic data only after two days of combined pevonedistat and ibrutinib treatment, therefore we were unable to completely exclude ibrutinib induction of CYP3A mediated pevonedistat metabolism with ongoing therapy.

The study is limited by small sample size at this time and short follow-up. The CLL/PLL cohort, in particular, has only four patients and thus conclusions of efficacy in this cohort are limited. In addition, at the time of study conception, in an effort to design a time limited therapeutic regimen, pevonedistat administration was limited to 8 cycles and ibrutinib to 18 cycles with an option to continue ibrutinib beyond the designated time period per physician discretion. Considering the good safety profile of the combination so far and the short PFS despite good response rates, the optimal duration of this combination therapy needs to be re-evaluated.

Confirmation of efficacy would require a larger phase 2 study in the R/R setting. Future studies could also evaluate this combination in the frontline setting particularly in CLL patients and high-risk MCL patients (those with *TP53* aberrations and/or complex karyotype) [39]. Adding a third drug such as venetoclax, which has demonstrated significant activity in both MCL [40] and CLL [41], could be considered to improve depth and durability of responses, with the caveat that toxicities would increase. Another consideration would be to evaluate the combination of pevonedistat with “second-generation” BTK inhibitors (e.g., acalabrutinib, zanubrutinib) which may further mitigate the risks of bleeding and atrial fibrillation.

We have previously reported that NAE inhibition in CLL cells led to downregulation of the pro-survival NFκB signaling, accompanied by rebalancing of the Bcl-2 family members in favor of apoptosis [6, 7, 42]. In stromal conditions mimicking the lymph node microenvironment, this led to reversal of drug resistance. Similar downstream effects of pevonedistat were observed in other lymphoid models, including activated B-cell type DLBCL cells [4]. Here, we used scRNA-Seq technology to confirm relevance of our preclinical findings in vivo, thus for the first time providing unique evidence of tumor-directed mechanistic effects of pevonedistat in patients. Pevonedistat rapidly (3 h) downregulated NFκB signaling in malignant B cells in patients with MCL. Additional pathways deregulated by pevonedistat were aligned with its known CRL targets, such as *CDKN1A* (p21) and HIF-1α. Thus, our work provides strong evidence for on-target effects of NAE inhibition in NHL.

We also have reported that pevonedistat modulates T cell immunity in patients with CLL, leading to decreased T_reg_ differentiation and a shift toward the T_H_1 phenotype, thereby exhibiting a strong potential to enhance anti-tumor immunity [43, 44]. Meanwhile, inhibitors of BCR-associated kinases also have strong immunomodulatory effects [45]. In particular, treatment with ibrutinib was shown to downregulate checkpoint inhibitors PD-1 and CTLA-4 in T cells, decrease the Treg/CD4 + T cell ratio as well as diminish the immunosuppressive properties of the CLL cells [46]. How combined use of pevonedistat and ibrutinib may alter immune cell function in patients with NHL and whether their effects can be employed in the future to harness anti-tumor immunity in other tumor types is a subject of ongoing investigations by our group.

In conclusion, this phase 1 study shows that the combination of fixed duration pevonedistat and continuous ibrutinib is safe and well tolerated in patients with R/R NHL without any new unexpected toxicities. The combination shows preliminary efficacy in R/R NHL, particularly in the patients with CLL and MCL. Larger trials are needed to establish the efficacy and optimal duration of therapy for this unique regimen.

## Supplementary information

Supplementary Methods

Supplementary Figures 1-2

Checklist

## Data Availability

Additional data pertaining to this clinical trial is available on clinicaltrials.gov under NCT03479268.
