# Supplementary material for: Pevonedistat, a Nedd8-activating enzyme inhibitor, in combination with ibrutinib in patients with relapsed/refractory B-cell non-Hodgkin lymphoma
Source: Blood Cancer J. 2023 Jan 11;13(1):9. doi: 10.1038/s41408-022-00763-w (PMC9834208; doi:10.1038/s41408-022-00763-w)
Supplement: Supplementary file 1 — Supplementary Methods [file 41408_2022_763_MOESM1_ESM.docx]

**Supplemental Methods**

*scRNA-seq*

For RNA analysis, peripheral blood was collected at baseline (prior to pevonedistat infusion on cycle 1 day 1) and 3 and 24 hours after pevonedistat infusion (the latter prior to the first dose of ibrutinib on cycle 1 day 2). Peripheral blood mononuclear cells (PBMCs) were isolated using standard Ficoll-Hypaque technique (Amersham). Red blood cells were lysed using ACK buffer (Thermo Fisher Scientific). PBMCs were viably frozen for future analysis.

*Single-cell library preparation and sequencing.* Upon thawing, PMBCs were washed and single cells were labeled with Cell Multiplexing Oligos (CMOs) (CellPlex Kit Set A, 1000261) (10X Genomics) according to the manufacturer’s instructions. Labeled samples were pooled in desired ratios. Cell concentration and viability of the pooled sample was determined using a TC20™ Automated Cell Counter (Bio-Rad). A total of 10,000 cells were targeted per pooled sample. Each sample pool was loaded into a different lane of a 10X chip (Chromium Next GEM Chip G Single Cell Kit, 1000127). cDNA libraries were generated using the Single Cell 3’ Library & Gel Bead kit version 3.1 (1000121). Indexed sequencing libraries were constructed using the reagents in the library Construction Kit (10xGenomics, 1000190). The barcode sequencing libraries were sequenced on the NovaSeq 6000 platform (Illumina) with paired-end sequencing and dual indexing. A total of 28, 10, 10 and 101 cycles were run for Read 1, i7 index, i5 index and Read 2, respectively. Raw sequences were processed and aligned to the GRChg38 genome using 10x Genomics’ CellRanger v6.1.1 “multi” pipeline with default settings. These experiments were performed in the Integrative Genomics and Bioinformatics Core and supported by the National Cancer Institute of the National Institutes of Health under award number P30CA033572.

*scRNA-seq data processing.* The sequenced reads were mapped to the GRCh38 human reference genome and the unique molecule identifier (UMIs) for each gene in each cell was counted by the Cell Ranger (10x Genomics). The resulting gene expression matrices were loaded into R (version 4.0.3) and analyzed with the Seurat pipeline (version 4.1.0) ^1^. Genes had to be expressed in at least three cells to be considered for analysis. Cells were filtered to retain those that contained at least 500 minimum unique genes expressed, no more than 5000 unique genes, more than 500 total UMIs and less than 10% of counts mapped to the mitochondrial genome. For the multiplexed samples from the same patient, we adopted the demultiplexing with hashtag oligos (HTOs) in the expression matrix by the function HTODemux implemented in Seurat with default parameters. Doublets were further removed using DoubletFinder (v2.0.3) with default parameters and a doublet rate threshold of 4% ^2^. For the remaining cells of 3 samples in each patient, the function “SCTransform” in Seurat was used to normalize data, scale data, and find variable features. After that, the 30 most informative principal components (PCA) of the selected top variable genes were used for cluster and visualize all cell types. Clusters were identified by the FindClusters function with a resolution of 0.1 and visualized using the Uniform Manifold Approximation and Projection (UMAP) for dimension reduction ^3^. The cell type of each cluster was annotated by SingleR (v1.8.1) following the standard procedure using the human PrimaryCell Atlas as the reference. The differential expressed genes (DEGs) analysis of each cluster was calculated by the FindAllMarkers function in Seurat with default parameters. Within each cluster, the DEGs between two time points were calculated by FindMarkers in Seurat. Here, the default parameters for both FindAllMarkers and FindMarkers in Seurat were Wilcoxon rank-sum test (two-sided), 0.25 for the log2 fold change cutoff and 0.10 for the parameter 'min.pct'.

*Copy number alteration analysis from single-cell RNA-seq.* InferCNV^4^ (v1.6.0) with the default parameters was used to predict the segmented copy-number alterations (CNAs) in scRNA-seq data. The B-cells selected from the pbmc3k dataset of a healthy subject in the SeuratData (0.2.1) were used as reference controls.

*Pathway analysis in single-cell RNA-seq.* For each cell, we calculated the enrichment scores of the pathways in the MSigDB hallmark gene sets (v7.2) using the AddModuleScore function in the Seurat package ^5^. The significantly enriched pathways between two conditions within each cluster were called by the Wilcoxon signed-rank test (two-sided) with an adjusted p-value less than 0.05.

**Supplemental Figure Legends.**

**Supplemental Figure 1. scRNA-seq profiling of immune cells in MCL (PV14).** *(A)* UMAP visualization of all immune cells colored by timepoint. Numbers in the parentheses show the cell numbers of conditions. *(B)* InferCNV analysis of B-cell clusters with normal B-cells as reference. *(C) SOX11* mRNA transcript expression across three B-cell clusters.

**Supplemental Figure 2. scRNA-seq landscape in MCL (PV24).** *(A)* UMAP visualization of all immune cells colored by immune cell type. *(B)* UMAP visualization of all cells colored by timepoint. Numbers in the parentheses show the cell numbers of conditions. *(C)* UMAP visualization of B-cells colored by timepoint. Numbers in the parentheses show the cell numbers of conditions.(D) The dotplot of all the signature genes at the 24-hour timepoint. (E) Violin plot shows the comparisons of enrichment scores of the HALLMARK_TNFA_SIGNALING_VIA_NFKB pathway in the three time points of the B-cells in patient PV24. The p-values were calculated by the Wilcoxon signed-rank test (two-sided).

**Supplemental Table 1**. **Study Treatment Dose Escalation Levels**

| **Dose Level** | **Pevonedistat (Cycles 1-8)**  intravenously on days 1, 3 and 5  Each cycle is 21 days | **Ibrutinib (Cycles 1-18)**  po daily starting C1D2  Each cycle is 21 days |
| --- | --- | --- |
| -1 | 10 mg/m^2^ | 420 (560) mg |
| 1 | 15 mg/m^2^ | 420 (560) mg |
| 2 | 20 mg/m^2^ | 420 (560) mg |
| 3 | 25 mg/m^2^ | 420 (560) mg |
| 4 | 37.5 mg/m^2^ | 420 (560) mg |
| 5 | 50 mg/m^2^ | 420 (560) mg |
| Patients with MCL received 560 mg ibrutinib. All others received 420 mg ibrutinib | | |

**Supplemental Table 2. List of Therapies by Lymphoma Subtype**

| **Patient** | **Lymphoma subtype** | **Prior therapies** |
| --- | --- | --- |
| 1 | DLBCL | R-CHOP -> tirabrutinib+idelalisib |
| 2 | DLBCL | R-CHOP -> BR |
| 3 | DLBCL | R-DA-EPOCH |
| 4 | DLBCL | R-CHOP |
| 5 | CLL | Benda-Obinutuzumab-> Entospletinib-Obinutuzumab |
| 6 | CLL | FCR - > Idelalisib/Ofatumumab -> Entospletinib-Obinutuzumab |
| 7 | CLL | FCR |
| 8 | MCL | BR |
| 9 | MCL | BR |
| 10 | MCL | BR |
| 11 | MCL | Ofatumumab-HyperCVAD followed by consolidative autoSCT with BuCy prep |
| 12 | MCL | BR |
| 13 | MCL | Rituximab-HyperCVAD followed by consolidative autoSCT with BuCy prep followed by maintenance rituximab |
| 14 | MCL | Rituximab-HyperCVAD followed by consolidative autoSCT with BuCy prep followed by maintenance rituximab |
| 15 | MCL | BR |
| 16 | FL | R-CVP- Radio immunotherapy |
| 17 | B-PLL | Rituximab |
| 18 | MZL | BR |

DLBCL- Diffuse large B-cell lymphoma, CLL- chronic lymphocytic leukemia, MCL- mantle cell lymphoma, FL- follicular lymphoma, B-PLL- B-prolymphocytic leukemia, MZL- marginal zone lymphoma, R-CHOP- rituximab-cyclophosphamide, doxorubicin, vincristine, prednisone, BR- bendamustine-rituximab, R-DA-EPOCH- rituximab-dose adjusted etoposide, prednisone, vincristine, cyclophosphamide, doxorubicin, FCR- fludarabine-cyclophosphamide-rituximab, HyperCVAD- Hyper fractionated cyclophosphamide, vincristine, doxorubicin, dexamethasone (course A), methotrexate and cytarabine (course B), autoSCT- autologous stem cell transplant, BuCy- Busulfan-cyclophosphamide, R-CVP- rituximab-cyclophosphamide-vincristine-prednisone

**Supplemental Table 3. Characteristics of Patients who Completed Study Treatment**

| **Patient** | **Diagnosis** | **Disease Characteristics** |
| --- | --- | --- |
| 1 | MCL | 42,add(X)(p22.3),-Y,der(2)t(2;15)(p23;q11.2),-8,add(10)(p11.2),-13,-15,add(17)(p13),add(18)(p11.2),-20,-22,+mar1,+mar2[10]/46,XY[15]. FISH showed del17p |
| 2 | B-PLL | Stemline:  46,XX,del(13)(q14.1q32),der(17;22)(q10;q10),add(18)(q23),+mar[1]  Sideline:  45,sl,der(5)t(5;13)(p14;q21.1),-13[4]  FISH: Positive for homozygous and heterozygous loss of D13S319, heterozygous loss of *TP53* |
| 3 | MCL | Normal karyotype. *TP53* wildtype |
| 4 | DLBCL | non-GCB subtype, double expressor |

MCL- mantle cell lymphoma, B-PLL- B-prolymphocytic leukemia, DLBCL- Diffuse large B-cell lymphoma

**Supplemental Table 4. DLBCL Patient Characteristics**

| **Patient** | **COO** | **MYC/BCL2/BCL6 status** | **Previous treatments** | **Response to doublet** |
| --- | --- | --- | --- | --- |
| 1 | non-GCB | - | RCHOP, Tirabrutinib + idelalisib | Stable disease |
| 2 | GCB | Double hit (MYC/BCL2) | RCHOP, BR | Response unknown (came off study due to toxicity) |
| 3 | non-GCB | Double expressor (MYC, BCL2) | DA-R-EPOCH with IT methotrexate | Complete response (maintained for over 3 years); ibrutinib stopped due to worsening dementia and declining quality of life |
| 4 | non-GCB | BCL-6 rearrangement | RCHOP | Progressive disease |

R-CHOP- rituximab-cyclophosphamide, doxorubicin, vincristine, prednisone, BR- bendamustine-rituximab, R-DA-EPOCH- rituximab-dose adjusted etoposide, prednisone, vincristine, cyclophosphamide, doxorubicin, IT- intrathecal

**Supplemental Table 5. Median (range) Pevonedistat Pharmacokinetic Parameters for Days 1 and 3**

| **Dose Cohort Day 1** | **Dose (mg)** | **T_max_**  **(h)** | **C_max_**  **(ng/mL)** | **T_½_**  **(h)** | **AUC _(0-inf)_**  **(ng.h/mL)** | **Clearance**  **(L/h)** | **Volume of distribution**  **Vd _(area)_ (L)** |
| --- | --- | --- | --- | --- | --- | --- | --- |
| **15 mg/m^2^**  **(n=3)** | 30  (27-33) | 1.05  (1.03-1.08) | 129.0 (63.6-143.0) | 6.9 (6.4-7.1) | 919.1 (671.6-1275.3) | 35.9 (23.5-40.2) | 329.9 (239.5-401.8) |
| **20 mg/m^2^ (n=3)** | 43.2 (41.-50.2) | 1.07 (1.03-1.08 | 94.1 (72.2-127.0) | 6.7 (6.3-6.7) | 912.5 (686.0-1021.9) | 49.1 (47.3-59.8) | 475.1 (459.1-540.6) |
| **25 mg/m^2^ (n=3)** | 45 (42-49) | 1.05 (1.0-1.017) | 218.0 (183.0-318.0) | 6.1 (5.7-12.1) | 1983.8 (1669.2-2016.3) | 22.3 (21.2-29.4) | 196.2 (172.5-510.7) |
| **37.5 mg/m^2^ (n=2)** | 74 (72-76) | 1.0  (1.0) | 211.5 (208.0-215.0) | 6.4 (5.6-7.2) | 1804.1 (1630.6-1977.5) | 38.4 (35.3-41.5) | 349.9 (334.5-365.3) |
| **50 mg/m^2^ (n=6)** | 102.5 (82-120) | 1.0 (0.5-2.0) | 385.0 (277.0-676.0) | 7.1 (4.9-9.0) | 3073.3 (2081.1-3370.2) | 33.1 (27.8-40.4) | 305.7 (271.2-526.8) |
|  |  |  |  |  |  |  |  |
| **Dose Cohort Day 3** | **Dose (mg)** | **T_max_**  **(h)** | **C_max_**  **(ng/mL)** | **T_½_**  **(h)** | **AUC _(0-inf)_**  **(ng.h/mL)** | **Clearance**  **(L/h)** | **Volume of distribution**  **Vd _(area)_ (L)** |
| **15 mg/m^2^**  **(n=3)** | 30  (27-33) | 1.05 (1.05-2.08) | 132.0 (124.0-167.0) | 5.6 (3.8-6.4) | 970.0 (741.8-1068.1) | 28.1. (27.8-44.5) | 246.6. (225.1-255.1) |
| **20 mg/m^2^ (n=3)** | 43.2 (41.-50.2) | 1.12 (1.08-1.17) | 106.0 (69.9-185.0) | 6.9 (6.8-9.9) | 1128.6 (595.6-1326.7) | 73.6 (44.5-325.6) | 733.2 (439.5-4625.9) |
| **25 mg/m^2^ (n=3)** | 45 (42-49) | 1.0  (1.0-2.25) | 249.0 (127.0-326.0) | 5.8 (5.5-11.0) | 1511.2 (1352.2 -2141.2) | 27.8 (21.0-36.2) | 231.9 (165.9-576.5) |
| **37.5 mg/m^2^ (n=2)** | 74 (72-76) | 1.0 (1.0) | 241.5  (218.0-265.0) | 6.8 (6.5-7.2) | 1793.3 (1744.5-1842.0) | 41.3  (41.3) | 405.0 (384.5-425.5 ) |
| **50 mg/m^2^ (n=6)** | 102.5 (82-120) | 1.0 (0.98-1.08) | 482.0 (416.0-574.0) | 6.1 (3.3-7.4) | 3196.1 (2053.6-3652.6. | 31.6 ( 26.1-51.1) | 239.6 (223.7-391.7) |

**Supplemental Table 6. Samples from patients submitted for RNA-Seq.**

| Sample name | Diagnosis | Method | Dose of pevonedistat |
| --- | --- | --- | --- |
| PV7 | DLBCL | scRNA-Seq | 25 mg/m^2^ |
| PV14 | MCL | scRNA-Seq | 37.5 mg/m^2^ |
| PV23 | MCL | scRNA-Seq | 50 mg/m^2^ |
| PV24 | MCL | scRNA-Seq | 50 mg/m^2^ |

**Supplemental Table 7. Gene expression in 3 distinct B-cell clusters in sample PV14.**

|  | p_val | avg_log2FC | pct.1 | pct.2 | p_val_adj | cluster |
| --- | --- | --- | --- | --- | --- | --- |
| ITGA4 | 0 | -0.76811017 | 0.01 | 0.431 | 0 | B-cell_0 |
| EEF1A1 | 0 | -0.84328474 | 1 | 1 | 0 | B-cell_0 |
| RPL35 | 2.66E-307 | -0.809111 | 1 | 1 | 4.67E-303 | B-cell_0 |
| SERPINF1 | 4.16E-263 | 1.103777799 | 0.665 | 0.061 | 7.30E-259 | B-cell_0 |
| RPL7A | 3.82E-260 | -0.76257879 | 0.998 | 1 | 6.72E-256 | B-cell_0 |
| MS4A1 | 5.40E-253 | -0.99778287 | 0.975 | 0.99 | 9.48E-249 | B-cell_0 |
| CD69 | 4.31E-229 | -1.16935443 | 0.224 | 0.691 | 7.57E-225 | B-cell_0 |
| RPL12 | 3.27E-225 | -0.75145206 | 0.997 | 1 | 5.75E-221 | B-cell_0 |
| TTN | 2.25E-206 | 1.38084854 | 0.725 | 0.214 | 3.95E-202 | B-cell_0 |
| CYBB | 1.29E-193 | -0.75285735 | 0.133 | 0.557 | 2.26E-189 | B-cell_0 |
| APOBEC3H | 4.96E-191 | 0.936161763 | 0.519 | 0.027 | 8.71E-187 | B-cell_0 |
| RRBP1 | 2.19E-180 | 1.063756055 | 0.751 | 0.309 | 3.85E-176 | B-cell_0 |
| IGHM | 3.35E-173 | 0.760134828 | 0.999 | 0.987 | 5.89E-169 | B-cell_0 |
| SET | 1.14E-170 | -0.81264761 | 0.784 | 0.95 | 2.00E-166 | B-cell_0 |
| NEFL | 7.86E-157 | 0.87111826 | 0.733 | 0.293 | 1.38E-152 | B-cell_0 |
| RPS11 | 1.07E-156 | -0.49257775 | 0.999 | 0.999 | 1.88E-152 | B-cell_0 |
| RPS6 | 8.29E-149 | -0.59056337 | 1 | 1 | 1.46E-144 | B-cell_0 |
| RPS27A | 3.49E-144 | 0.402771139 | 1 | 1 | 6.13E-140 | B-cell_0 |
| FCRL1 | 6.67E-142 | -0.75733393 | 0.252 | 0.615 | 1.17E-137 | B-cell_0 |
| RPLP1 | 4.42E-137 | 0.41749897 | 1 | 1 | 7.77E-133 | B-cell_0 |
| AMN | 1.02E-133 | 0.676311136 | 0.51 | 0.107 | 1.79E-129 | B-cell_0 |
| B2M | 7.53E-126 | 0.39701775 | 1 | 1 | 1.32E-121 | B-cell_0 |
| PMEPA1 | 1.21E-122 | -0.31722602 | 0.002 | 0.169 | 2.12E-118 | B-cell_0 |
| TSHZ2 | 1.47E-122 | 0.719253823 | 0.821 | 0.453 | 2.58E-118 | B-cell_0 |
| AIM2 | 9.93E-122 | 0.742195844 | 0.783 | 0.454 | 1.74E-117 | B-cell_0 |
| SYK | 1.47E-121 | -0.63129655 | 0.183 | 0.506 | 2.58E-117 | B-cell_0 |
| CDKN2A | 5.49E-120 | -0.28748314 | 0.004 | 0.174 | 9.64E-116 | B-cell_0 |
| LTB | 1.70E-119 | 0.647484563 | 0.995 | 0.976 | 2.99E-115 | B-cell_0 |
| AIRE | 2.67E-119 | 0.80424513 | 0.359 | 0.013 | 4.69E-115 | B-cell_0 |
| CCDC141 | 7.43E-116 | 0.669727251 | 0.438 | 0.074 | 1.30E-111 | B-cell_0 |
| FCRL2 | 7.60E-116 | -0.73978088 | 0.414 | 0.722 | 1.33E-111 | B-cell_0 |
| JCHAIN | 1.08E-111 | 0.50746282 | 0.408 | 0.059 | 1.90E-107 | B-cell_0 |
| RPL21 | 8.50E-111 | -0.56273203 | 1 | 1 | 1.49E-106 | B-cell_0 |
| MS4A7 | 2.93E-104 | -0.37140449 | 0.031 | 0.233 | 5.14E-100 | B-cell_0 |
| RPS21 | 1.60E-103 | 0.332328932 | 1 | 1 | 2.81E-99 | B-cell_0 |
| WNT3 | 1.80E-102 | 0.718391171 | 0.564 | 0.213 | 3.16E-98 | B-cell_0 |
| TXNIP | 1.54E-98 | 0.607482874 | 0.992 | 0.965 | 2.71E-94 | B-cell_0 |
| RPL23A | 5.69E-97 | 0.333539629 | 1 | 1 | 9.99E-93 | B-cell_0 |
| GADD45B | 1.07E-95 | 0.766406478 | 0.691 | 0.38 | 1.89E-91 | B-cell_0 |
| RPS24 | 2.43E-95 | 0.326720218 | 1 | 1 | 4.26E-91 | B-cell_0 |
| RPL41 | 3.75E-94 | 0.306928985 | 1 | 1 | 6.59E-90 | B-cell_0 |
| RPS7 | 3.85E-93 | 0.374028087 | 1 | 1 | 6.75E-89 | B-cell_0 |
| HRK | 1.02E-91 | 0.596928337 | 0.48 | 0.141 | 1.79E-87 | B-cell_0 |
| IGLC3 | 6.20E-90 | 0.404744023 | 1 | 0.987 | 1.09E-85 | B-cell_0 |
| S100A4 | 1.91E-89 | 0.618487293 | 0.983 | 0.939 | 3.35E-85 | B-cell_0 |
| RPL27A | 1.81E-88 | 0.289621747 | 1 | 1 | 3.17E-84 | B-cell_0 |
| SRP14 | 9.55E-86 | -0.50921058 | 0.846 | 0.925 | 1.68E-81 | B-cell_0 |
| MCTP2 | 7.84E-85 | -0.48879701 | 0.202 | 0.476 | 1.38E-80 | B-cell_0 |
| FNBP1 | 2.19E-84 | -0.60795311 | 0.532 | 0.755 | 3.85E-80 | B-cell_0 |
| SOX11 | 8.89E-81 | -0.47499246 | 0.115 | 0.355 | 1.56E-76 | B-cell_0 |
| AC133644.2 | 2.55E-80 | 0.45371194 | 0.271 | 0.016 | 4.48E-76 | B-cell_0 |
| RPL19 | 3.98E-80 | 0.288170926 | 1 | 1 | 7.00E-76 | B-cell_0 |
| RPL24 | 4.38E-79 | -0.39410272 | 1 | 1 | 7.70E-75 | B-cell_0 |
| BMPR2 | 1.47E-78 | -0.26529202 | 0.025 | 0.183 | 2.59E-74 | B-cell_0 |
| LGALS3 | 3.33E-78 | 0.539188195 | 0.471 | 0.173 | 5.85E-74 | B-cell_0 |
| FCMR | 2.14E-77 | -0.53115013 | 0.873 | 0.948 | 3.77E-73 | B-cell_0 |
| EBLN3P | 3.94E-75 | -0.58244648 | 0.51 | 0.722 | 6.92E-71 | B-cell_0 |
| EPCAM | 1.80E-74 | 0.480396278 | 0.495 | 0.193 | 3.16E-70 | B-cell_0 |
| ZNF804A | 1.10E-73 | 0.64158848 | 0.565 | 0.279 | 1.94E-69 | B-cell_0 |
| APRT | 1.17E-73 | 0.504076837 | 0.793 | 0.56 | 2.06E-69 | B-cell_0 |
| HCST | 1.33E-73 | 0.481229685 | 0.39 | 0.106 | 2.33E-69 | B-cell_0 |
| AC253572.2 | 3.51E-73 | -0.84081936 | 0.426 | 0.67 | 6.17E-69 | B-cell_0 |
| CCDC151 | 1.29E-72 | 0.495423525 | 0.491 | 0.188 | 2.26E-68 | B-cell_0 |
| CD74 | 3.59E-71 | 0.269572695 | 1 | 1 | 6.30E-67 | B-cell_0 |
| KLF13 | 3.76E-71 | -0.51606724 | 0.321 | 0.569 | 6.60E-67 | B-cell_0 |
| SOD1 | 2.96E-70 | 0.452549687 | 0.917 | 0.778 | 5.19E-66 | B-cell_0 |
| EBF1 | 3.79E-70 | -0.49241921 | 0.263 | 0.513 | 6.66E-66 | B-cell_0 |
| TOMM7 | 5.57E-70 | 0.383656646 | 0.997 | 0.993 | 9.79E-66 | B-cell_0 |
| CXCR4 | 1.27E-69 | -0.82750463 | 0.56 | 0.734 | 2.22E-65 | B-cell_0 |
| TMSB4X | 1.40E-68 | 0.337793678 | 1 | 1 | 2.46E-64 | B-cell_0 |
| PCDH9 | 2.44E-68 | 0.496378231 | 0.289 | 0.045 | 4.29E-64 | B-cell_0 |
| SETX | 1.47E-66 | -0.56289431 | 0.374 | 0.609 | 2.58E-62 | B-cell_0 |
| SERF2 | 2.70E-66 | 0.362106627 | 0.999 | 0.99 | 4.73E-62 | B-cell_0 |
| CNTRL | 5.32E-66 | -0.49287934 | 0.341 | 0.59 | 9.34E-62 | B-cell_0 |
| IQSEC1 | 2.49E-64 | -0.46243901 | 0.214 | 0.45 | 4.38E-60 | B-cell_0 |
| HNRNPK | 1.88E-63 | -0.48304093 | 0.658 | 0.799 | 3.31E-59 | B-cell_0 |
| EPB41 | 5.34E-63 | 0.484243112 | 0.889 | 0.753 | 9.38E-59 | B-cell_0 |
| MPHOSPH8 | 8.05E-62 | -0.60205018 | 0.414 | 0.623 | 1.41E-57 | B-cell_0 |
| TCF7 | 1.46E-61 | 0.478226318 | 0.483 | 0.216 | 2.56E-57 | B-cell_0 |
| HLA-B | 2.04E-61 | 0.258343975 | 1 | 1 | 3.58E-57 | B-cell_0 |
| RALGPS2 | 3.02E-61 | -0.43703101 | 0.945 | 0.975 | 5.30E-57 | B-cell_0 |
| APOD | 1.46E-60 | 0.478692406 | 0.316 | 0.078 | 2.56E-56 | B-cell_0 |
| P2RX5 | 2.60E-60 | -0.5068833 | 0.372 | 0.599 | 4.57E-56 | B-cell_0 |
| PABPC1 | 5.33E-60 | -0.35397355 | 0.99 | 0.997 | 9.36E-56 | B-cell_0 |
| BTG1 | 6.80E-60 | -0.43395783 | 0.943 | 0.972 | 1.19E-55 | B-cell_0 |
| COX7A2 | 9.66E-60 | -0.45828712 | 0.496 | 0.692 | 1.70E-55 | B-cell_0 |
| APPL1 | 2.34E-59 | 0.459482856 | 0.819 | 0.598 | 4.12E-55 | B-cell_0 |
| SMPD3 | 3.26E-59 | 0.449420794 | 0.412 | 0.164 | 5.72E-55 | B-cell_0 |
| BTLA | 5.32E-59 | -0.2620427 | 0.037 | 0.179 | 9.34E-55 | B-cell_0 |
| MEF2A | 6.77E-59 | -0.42738675 | 0.262 | 0.495 | 1.19E-54 | B-cell_0 |
| HERC2 | 8.81E-59 | -0.49178237 | 0.357 | 0.58 | 1.55E-54 | B-cell_0 |
| ZBTB20 | 2.81E-58 | -0.5423641 | 0.622 | 0.772 | 4.93E-54 | B-cell_0 |
| EIF1 | 3.75E-58 | 0.277141909 | 1 | 1 | 6.58E-54 | B-cell_0 |
| N4BP2L2 | 2.82E-57 | -0.47156591 | 0.628 | 0.784 | 4.95E-53 | B-cell_0 |
| CD24 | 3.82E-57 | 0.492065186 | 0.672 | 0.427 | 6.71E-53 | B-cell_0 |
| CRIP2 | 2.24E-56 | 0.469380577 | 0.663 | 0.401 | 3.92E-52 | B-cell_0 |
| RPL14 | 4.31E-55 | 0.28270352 | 1 | 1 | 7.57E-51 | B-cell_0 |
| HLA-A | 2.59E-54 | 0.280830643 | 1 | 1 | 4.54E-50 | B-cell_0 |
| HNRNPU | 3.12E-54 | -0.4619345 | 0.848 | 0.922 | 5.49E-50 | B-cell_0 |
| RPL39 | 5.96E-54 | -0.28030206 | 1 | 1 | 1.05E-49 | B-cell_0 |
| MT-CO1 | 6.46E-54 | 0.270689099 | 0.999 | 1 | 1.13E-49 | B-cell_0 |
| AC103591.3 | 1.37E-53 | -0.62658832 | 0.144 | 0.339 | 2.41E-49 | B-cell_0 |
| PAX5 | 1.50E-53 | -0.4747668 | 0.756 | 0.847 | 2.63E-49 | B-cell_0 |
| RPL10 | 2.30E-53 | 0.25101712 | 1 | 1 | 4.03E-49 | B-cell_0 |
| AC245014.3 | 1.57E-52 | -0.58626361 | 0.173 | 0.374 | 2.76E-48 | B-cell_0 |
| RAPGEF1 | 2.75E-52 | -0.36223476 | 0.2 | 0.412 | 4.82E-48 | B-cell_0 |
| FAM214A | 2.90E-52 | -0.37315768 | 0.193 | 0.403 | 5.10E-48 | B-cell_0 |
| SNX22 | 3.51E-52 | -0.28551482 | 0.077 | 0.242 | 6.16E-48 | B-cell_0 |
| SERPINB6 | 6.92E-52 | 0.346985072 | 0.306 | 0.087 | 1.22E-47 | B-cell_0 |
| TNFRSF1B | 6.49E-51 | 0.346406006 | 0.321 | 0.1 | 1.14E-46 | B-cell_0 |
| PARP14 | 1.06E-50 | -0.50683704 | 0.373 | 0.579 | 1.86E-46 | B-cell_0 |
| SSBP2 | 2.18E-50 | 0.305680895 | 0.241 | 0.046 | 3.82E-46 | B-cell_0 |
| GOLGA8B | 2.24E-50 | -0.41567121 | 0.273 | 0.482 | 3.93E-46 | B-cell_0 |
| UBE3A | 2.59E-50 | -0.38008667 | 0.214 | 0.424 | 4.56E-46 | B-cell_0 |
| RPL35A | 5.69E-50 | -0.28080792 | 1 | 1 | 9.98E-46 | B-cell_0 |
| GAPT | 7.13E-50 | -0.35228667 | 0.138 | 0.326 | 1.25E-45 | B-cell_0 |
| LSM10 | 1.09E-49 | 0.411301959 | 0.574 | 0.348 | 1.92E-45 | B-cell_0 |
| RSL24D1 | 1.93E-49 | -0.41733406 | 0.342 | 0.549 | 3.38E-45 | B-cell_0 |
| SHTN1 | 8.98E-49 | 0.280019184 | 0.188 | 0.016 | 1.58E-44 | B-cell_0 |
| CCDC50 | 9.94E-49 | -0.38941313 | 0.866 | 0.914 | 1.75E-44 | B-cell_0 |
| ATP6V1G1 | 2.62E-48 | -0.40928624 | 0.561 | 0.729 | 4.60E-44 | B-cell_0 |
| STMN1 | 1.57E-47 | -0.42752274 | 0.19 | 0.385 | 2.75E-43 | B-cell_0 |
| CD27 | 1.96E-47 | 0.416054063 | 0.633 | 0.408 | 3.44E-43 | B-cell_0 |
| NSMCE3 | 1.77E-46 | -0.37999547 | 0.28 | 0.487 | 3.11E-42 | B-cell_0 |
| CBLB | 2.48E-46 | -0.34307327 | 0.204 | 0.405 | 4.36E-42 | B-cell_0 |
| B4GALT1 | 1.74E-45 | -0.37470964 | 0.25 | 0.449 | 3.06E-41 | B-cell_0 |
| PSIP1 | 9.64E-45 | -0.43290841 | 0.422 | 0.611 | 1.69E-40 | B-cell_0 |
| ROMO1 | 1.34E-44 | 0.380295032 | 0.716 | 0.509 | 2.35E-40 | B-cell_0 |
| CRIP1 | 5.10E-44 | 0.414589591 | 0.989 | 0.966 | 8.95E-40 | B-cell_0 |
| SQSTM1 | 5.14E-44 | 0.423072229 | 0.682 | 0.475 | 9.03E-40 | B-cell_0 |
| TUT7 | 1.04E-43 | -0.33981174 | 0.16 | 0.34 | 1.83E-39 | B-cell_0 |
| APOBEC3G | 2.67E-43 | 0.398747506 | 0.598 | 0.385 | 4.69E-39 | B-cell_0 |
| UGCG | 4.45E-43 | -0.35043724 | 0.216 | 0.406 | 7.82E-39 | B-cell_0 |
| HMGB1 | 6.27E-43 | -0.3880731 | 0.913 | 0.945 | 1.10E-38 | B-cell_0 |
| IFNG-AS1 | 9.73E-43 | 0.335208702 | 0.404 | 0.183 | 1.71E-38 | B-cell_0 |
| RPS17 | 1.86E-42 | -0.28010083 | 0.997 | 0.998 | 3.27E-38 | B-cell_0 |
| HMCES | 2.19E-42 | -0.25819388 | 0.091 | 0.244 | 3.84E-38 | B-cell_0 |
| GNG7 | 4.11E-42 | 0.305895012 | 0.339 | 0.129 | 7.21E-38 | B-cell_0 |
| CCND1 | 4.72E-42 | 0.495251472 | 0.924 | 0.819 | 8.29E-38 | B-cell_0 |
| ZMYM2 | 3.83E-41 | -0.33763111 | 0.183 | 0.362 | 6.72E-37 | B-cell_0 |
| SP100 | 4.43E-41 | -0.38387052 | 0.914 | 0.951 | 7.77E-37 | B-cell_0 |
| LINC00926 | 6.75E-41 | -0.40770062 | 0.791 | 0.859 | 1.19E-36 | B-cell_0 |
| SHISA8 | 9.01E-41 | 0.281150582 | 0.231 | 0.058 | 1.58E-36 | B-cell_0 |
| SAV1 | 1.09E-40 | -0.2753404 | 0.112 | 0.271 | 1.92E-36 | B-cell_0 |
| RPL15 | 2.21E-40 | 0.254849735 | 1 | 1 | 3.88E-36 | B-cell_0 |
| NIBAN3 | 2.42E-40 | -0.40651766 | 0.761 | 0.847 | 4.25E-36 | B-cell_0 |
| ELOVL5 | 5.68E-40 | 0.356754397 | 0.678 | 0.479 | 9.97E-36 | B-cell_0 |
| RYR1 | 6.21E-40 | 0.268568174 | 0.231 | 0.061 | 1.09E-35 | B-cell_0 |
| UBE2R2 | 6.63E-40 | -0.31646348 | 0.208 | 0.391 | 1.16E-35 | B-cell_0 |
| ANP32B | 8.47E-40 | -0.39278852 | 0.576 | 0.72 | 1.49E-35 | B-cell_0 |
| DSP | 1.19E-39 | -0.27363683 | 0.042 | 0.155 | 2.09E-35 | B-cell_0 |
| RASSF6 | 1.29E-39 | 0.484426485 | 0.302 | 0.114 | 2.26E-35 | B-cell_0 |
| GNB5 | 1.56E-39 | -0.30833605 | 0.163 | 0.334 | 2.73E-35 | B-cell_0 |
| CD52 | 1.71E-39 | 0.259539666 | 1 | 0.999 | 3.01E-35 | B-cell_0 |
| PPIB | 2.48E-39 | 0.308177596 | 0.822 | 0.648 | 4.36E-35 | B-cell_0 |
| ACSM3 | 1.18E-38 | -0.29091663 | 0.093 | 0.235 | 2.07E-34 | B-cell_0 |
| CIRBP | 2.22E-38 | 0.309130886 | 0.966 | 0.907 | 3.89E-34 | B-cell_0 |
| SERP1 | 5.04E-38 | -0.36146081 | 0.739 | 0.831 | 8.85E-34 | B-cell_0 |
| HLA-C | 9.09E-38 | 0.251036428 | 0.998 | 0.996 | 1.60E-33 | B-cell_0 |
| SH3BP5 | 1.83E-37 | 0.326081543 | 0.887 | 0.757 | 3.21E-33 | B-cell_0 |
| SECISBP2 | 2.95E-37 | -0.33510627 | 0.255 | 0.435 | 5.18E-33 | B-cell_0 |
| RTF1 | 3.56E-37 | -0.35509482 | 0.301 | 0.489 | 6.25E-33 | B-cell_0 |
| CALR | 3.68E-37 | 0.44799222 | 0.734 | 0.572 | 6.46E-33 | B-cell_0 |
| PNRC1 | 3.82E-37 | -0.40652993 | 0.831 | 0.886 | 6.71E-33 | B-cell_0 |
| ACP5 | 4.07E-37 | 0.386280645 | 0.637 | 0.444 | 7.14E-33 | B-cell_0 |
| PRDX1 | 5.40E-37 | 0.346789574 | 0.729 | 0.548 | 9.47E-33 | B-cell_0 |
| POU2F2 | 5.87E-37 | -0.42333138 | 0.778 | 0.857 | 1.03E-32 | B-cell_0 |
| PTPRC | 1.09E-36 | -0.31031544 | 0.97 | 0.98 | 1.91E-32 | B-cell_0 |
| ICAM3 | 1.22E-36 | 0.33535386 | 0.696 | 0.509 | 2.15E-32 | B-cell_0 |
| OSTF1 | 1.31E-36 | -0.293753 | 0.185 | 0.352 | 2.30E-32 | B-cell_0 |
| TLN1 | 2.08E-36 | -0.39461033 | 0.438 | 0.599 | 3.64E-32 | B-cell_0 |
| ITPR2 | 2.37E-36 | -0.42773043 | 0.371 | 0.545 | 4.16E-32 | B-cell_0 |
| NME2 | 3.09E-36 | 0.31669752 | 0.977 | 0.94 | 5.42E-32 | B-cell_0 |
| GANC | 3.76E-36 | -0.2532128 | 0.116 | 0.265 | 6.60E-32 | B-cell_0 |
| PTBP3 | 5.29E-36 | -0.36840597 | 0.314 | 0.488 | 9.29E-32 | B-cell_0 |
| CEP170 | 5.33E-36 | -0.28469166 | 0.137 | 0.294 | 9.35E-32 | B-cell_0 |
| MT-ND3 | 6.50E-36 | -0.2509873 | 0.999 | 1 | 1.14E-31 | B-cell_0 |
| RAD23B | 9.10E-36 | -0.27656266 | 0.187 | 0.356 | 1.60E-31 | B-cell_0 |
| GPSM3 | 9.67E-36 | -0.36452448 | 0.496 | 0.648 | 1.70E-31 | B-cell_0 |
| HSP90B1 | 2.43E-35 | 0.335698011 | 0.771 | 0.608 | 4.28E-31 | B-cell_0 |
| ZCCHC7 | 5.02E-35 | -0.3732673 | 0.36 | 0.536 | 8.81E-31 | B-cell_0 |
| SAP18 | 7.30E-35 | -0.36656989 | 0.418 | 0.578 | 1.28E-30 | B-cell_0 |
| PHPT1 | 1.35E-34 | -0.25820152 | 0.135 | 0.289 | 2.37E-30 | B-cell_0 |
| CORO1B | 1.40E-34 | 0.351319639 | 0.629 | 0.437 | 2.45E-30 | B-cell_0 |
| SSBP3 | 1.93E-34 | 0.314363285 | 0.411 | 0.216 | 3.39E-30 | B-cell_0 |
| CKLF | 1.94E-34 | 0.323221303 | 0.459 | 0.266 | 3.41E-30 | B-cell_0 |
| CUTA | 2.45E-34 | 0.315299016 | 0.657 | 0.46 | 4.31E-30 | B-cell_0 |
| SENP6 | 2.58E-34 | -0.31442944 | 0.25 | 0.426 | 4.53E-30 | B-cell_0 |
| TUT4 | 3.14E-34 | -0.38801106 | 0.655 | 0.754 | 5.51E-30 | B-cell_0 |
| ZNF106 | 5.44E-34 | -0.30309791 | 0.196 | 0.361 | 9.56E-30 | B-cell_0 |
| IGHA1 | 7.70E-34 | -1.29716064 | 0.549 | 0.672 | 1.35E-29 | B-cell_0 |
| MAP1LC3A | 1.79E-33 | 0.263696239 | 0.192 | 0.048 | 3.14E-29 | B-cell_0 |
| CENPM | 4.42E-33 | 0.313576411 | 0.41 | 0.221 | 7.76E-29 | B-cell_0 |
| RPL22L1 | 5.46E-33 | -0.35206895 | 0.331 | 0.499 | 9.59E-29 | B-cell_0 |
| ELK4 | 8.99E-33 | -0.33942294 | 0.247 | 0.408 | 1.58E-28 | B-cell_0 |
| HCK | 1.47E-32 | -0.28162215 | 0.181 | 0.341 | 2.59E-28 | B-cell_0 |
| C16orf74 | 1.50E-32 | 0.304896997 | 0.489 | 0.294 | 2.64E-28 | B-cell_0 |
| JUN | 1.55E-32 | 0.344781711 | 0.993 | 0.981 | 2.71E-28 | B-cell_0 |
| MYO1E | 1.57E-32 | 0.326561479 | 0.544 | 0.355 | 2.76E-28 | B-cell_0 |
| TRA2B | 2.04E-32 | -0.26803624 | 0.958 | 0.981 | 3.59E-28 | B-cell_0 |
| TTF1 | 2.72E-32 | -0.25097088 | 0.109 | 0.246 | 4.78E-28 | B-cell_0 |
| EDF1 | 4.11E-32 | -0.32620895 | 0.652 | 0.752 | 7.22E-28 | B-cell_0 |
| NCR3 | 5.07E-32 | 0.331218847 | 0.544 | 0.352 | 8.90E-28 | B-cell_0 |
| BMP3 | 5.90E-32 | 0.284393689 | 0.277 | 0.111 | 1.04E-27 | B-cell_0 |
| UCP2 | 9.30E-32 | 0.314679614 | 0.966 | 0.931 | 1.63E-27 | B-cell_0 |
| VAMP8 | 9.73E-32 | 0.298675877 | 0.693 | 0.506 | 1.71E-27 | B-cell_0 |
| GOLGA8A | 1.34E-31 | -0.32016443 | 0.281 | 0.445 | 2.34E-27 | B-cell_0 |
| FOXP1 | 1.88E-31 | -0.33715704 | 0.934 | 0.939 | 3.31E-27 | B-cell_0 |
| OIP5-AS1 | 2.16E-31 | -0.32058972 | 0.285 | 0.446 | 3.79E-27 | B-cell_0 |
| ADTRP | 4.91E-31 | 0.265763704 | 0.29 | 0.123 | 8.63E-27 | B-cell_0 |
| KIAA2026 | 6.57E-31 | -0.30655301 | 0.266 | 0.436 | 1.15E-26 | B-cell_0 |
| STRBP | 7.51E-31 | -0.3667709 | 0.446 | 0.594 | 1.32E-26 | B-cell_0 |
| KATNBL1 | 8.03E-31 | -0.26296945 | 0.171 | 0.322 | 1.41E-26 | B-cell_0 |
| CCDC191 | 1.00E-30 | 0.285396781 | 0.565 | 0.36 | 1.76E-26 | B-cell_0 |
| ARPC1B | 1.18E-30 | 0.320649582 | 0.894 | 0.805 | 2.08E-26 | B-cell_0 |
| PALM2-AKAP2 | 1.76E-30 | -0.33161323 | 0.265 | 0.427 | 3.10E-26 | B-cell_0 |
| C9orf78 | 6.13E-30 | -0.32133848 | 0.316 | 0.469 | 1.08E-25 | B-cell_0 |
| ETS1 | 1.03E-29 | -0.37166711 | 0.702 | 0.786 | 1.80E-25 | B-cell_0 |
| CNTNAP2 | 1.48E-29 | 0.266345422 | 0.242 | 0.09 | 2.60E-25 | B-cell_0 |
| ATP5MPL | 2.03E-29 | 0.278475484 | 0.774 | 0.606 | 3.57E-25 | B-cell_0 |
| DNMBP | 2.17E-29 | 0.269842458 | 0.312 | 0.149 | 3.82E-25 | B-cell_0 |
| ZFAND5 | 2.36E-29 | -0.27411586 | 0.23 | 0.392 | 4.14E-25 | B-cell_0 |
| TFRC | 2.58E-29 | 0.312245558 | 0.476 | 0.294 | 4.53E-25 | B-cell_0 |
| FAM120AOS | 6.00E-29 | -0.27854583 | 0.217 | 0.372 | 1.05E-24 | B-cell_0 |
| PDIA3 | 7.47E-29 | 0.298577029 | 0.651 | 0.469 | 1.31E-24 | B-cell_0 |
| SKIL | 1.02E-28 | -0.36070687 | 0.278 | 0.432 | 1.78E-24 | B-cell_0 |
| ATRX | 1.09E-28 | -0.33483609 | 0.829 | 0.883 | 1.91E-24 | B-cell_0 |
| CAMK2D | 1.13E-28 | -0.31152044 | 0.233 | 0.386 | 1.98E-24 | B-cell_0 |
| TRAPPC5 | 1.19E-28 | 0.29670622 | 0.793 | 0.633 | 2.10E-24 | B-cell_0 |
| AKAP13 | 2.18E-28 | -0.27904508 | 0.891 | 0.935 | 3.82E-24 | B-cell_0 |
| RESF1 | 2.50E-28 | -0.37507909 | 0.785 | 0.85 | 4.39E-24 | B-cell_0 |
| CD47 | 6.25E-28 | -0.35168529 | 0.555 | 0.671 | 1.10E-23 | B-cell_0 |
| PHACTR1 | 1.02E-27 | -0.25432354 | 0.133 | 0.265 | 1.79E-23 | B-cell_0 |
| TCL1A | 1.07E-27 | -0.37760006 | 0.366 | 0.519 | 1.88E-23 | B-cell_0 |
| TPM3 | 1.14E-27 | -0.31422403 | 0.873 | 0.91 | 2.01E-23 | B-cell_0 |
| SMC5 | 1.23E-27 | -0.26356868 | 0.183 | 0.327 | 2.16E-23 | B-cell_0 |
| PRRC2B | 1.28E-27 | -0.2813639 | 0.267 | 0.423 | 2.24E-23 | B-cell_0 |
| FAM107B | 1.43E-27 | -0.37406769 | 0.565 | 0.675 | 2.50E-23 | B-cell_0 |
| SIAH2 | 7.81E-27 | -0.27359908 | 0.275 | 0.428 | 1.37E-22 | B-cell_0 |
| SMCHD1 | 9.85E-27 | -0.28487505 | 0.907 | 0.937 | 1.73E-22 | B-cell_0 |
| SCIMP | 1.21E-26 | 0.296333844 | 0.62 | 0.442 | 2.12E-22 | B-cell_0 |
| AC044849.1 | 2.10E-26 | 0.345122317 | 0.575 | 0.406 | 3.68E-22 | B-cell_0 |
| UTRN | 2.35E-26 | -0.36935775 | 0.418 | 0.559 | 4.13E-22 | B-cell_0 |
| HNRNPDL | 2.85E-26 | -0.29059219 | 0.848 | 0.895 | 5.00E-22 | B-cell_0 |
| ITGB1 | 2.90E-26 | 0.257903388 | 0.235 | 0.096 | 5.09E-22 | B-cell_0 |
| SLTM | 4.32E-26 | -0.36260878 | 0.468 | 0.605 | 7.59E-22 | B-cell_0 |
| FLNB | 4.34E-26 | 0.25878449 | 0.34 | 0.184 | 7.61E-22 | B-cell_0 |
| SPTLC2 | 5.27E-26 | 0.266534931 | 0.385 | 0.223 | 9.26E-22 | B-cell_0 |
| APOBEC3C | 5.85E-26 | 0.295048607 | 0.57 | 0.404 | 1.03E-21 | B-cell_0 |
| SENP7 | 1.53E-25 | -0.29756369 | 0.241 | 0.383 | 2.69E-21 | B-cell_0 |
| DHRS7 | 1.57E-25 | 0.278277317 | 0.575 | 0.41 | 2.76E-21 | B-cell_0 |
| SMC6 | 1.88E-25 | -0.32728918 | 0.366 | 0.51 | 3.31E-21 | B-cell_0 |
| SNHG8 | 2.11E-25 | 0.275364659 | 0.747 | 0.606 | 3.71E-21 | B-cell_0 |
| TMBIM6 | 2.62E-25 | 0.260109184 | 0.876 | 0.791 | 4.60E-21 | B-cell_0 |
| H1FX | 5.66E-25 | -0.42035789 | 0.819 | 0.908 | 9.93E-21 | B-cell_0 |
| COBLL1 | 9.44E-25 | 0.265887734 | 0.884 | 0.769 | 1.66E-20 | B-cell_0 |
| COX6C | 1.07E-24 | 0.255570972 | 0.911 | 0.82 | 1.87E-20 | B-cell_0 |
| SNHG7 | 2.41E-24 | -0.31054454 | 0.446 | 0.583 | 4.24E-20 | B-cell_0 |
| COX20 | 3.60E-24 | -0.29251652 | 0.36 | 0.501 | 6.32E-20 | B-cell_0 |
| CDK2AP2 | 4.34E-24 | 0.283144006 | 0.385 | 0.234 | 7.63E-20 | B-cell_0 |
| HNRNPA1 | 5.09E-24 | -0.27511741 | 0.946 | 0.963 | 8.94E-20 | B-cell_0 |
| WASHC4 | 5.42E-24 | -0.30205476 | 0.37 | 0.513 | 9.51E-20 | B-cell_0 |
| SPTAN1 | 1.54E-23 | -0.27586479 | 0.291 | 0.433 | 2.71E-19 | B-cell_0 |
| CDV3 | 1.71E-23 | -0.26838585 | 0.81 | 0.859 | 3.00E-19 | B-cell_0 |
| BCL11A | 3.84E-23 | -0.29576775 | 0.808 | 0.854 | 6.75E-19 | B-cell_0 |
| PLEKHA2 | 9.85E-23 | -0.30612089 | 0.656 | 0.742 | 1.73E-18 | B-cell_0 |
| FCRL3 | 2.26E-22 | -0.29150075 | 0.271 | 0.409 | 3.96E-18 | B-cell_0 |
| EHMT1 | 3.47E-22 | -0.27628557 | 0.324 | 0.461 | 6.08E-18 | B-cell_0 |
| MTSS1 | 8.94E-22 | 0.254992487 | 0.939 | 0.905 | 1.57E-17 | B-cell_0 |
| ADI1 | 9.28E-22 | 0.272492649 | 0.49 | 0.343 | 1.63E-17 | B-cell_0 |
| GTF2I | 1.08E-21 | -0.2832295 | 0.655 | 0.749 | 1.90E-17 | B-cell_0 |
| TRIM22 | 1.32E-21 | -0.30066014 | 0.388 | 0.513 | 2.31E-17 | B-cell_0 |
| ZNF107 | 1.61E-21 | -0.30777928 | 0.341 | 0.471 | 2.83E-17 | B-cell_0 |
| POLR1D | 1.85E-21 | -0.26053464 | 0.354 | 0.487 | 3.25E-17 | B-cell_0 |
| WIPF1 | 2.17E-21 | -0.26576265 | 0.322 | 0.458 | 3.82E-17 | B-cell_0 |
| RHOB | 7.85E-21 | 0.300527856 | 0.717 | 0.583 | 1.38E-16 | B-cell_0 |
| MBD4 | 1.41E-20 | -0.30167908 | 0.544 | 0.647 | 2.47E-16 | B-cell_0 |
| DHX36 | 1.63E-20 | -0.2831478 | 0.467 | 0.592 | 2.86E-16 | B-cell_0 |
| PEG10 | 2.05E-20 | 0.32852406 | 0.443 | 0.301 | 3.60E-16 | B-cell_0 |
| NUCKS1 | 2.17E-20 | -0.29511382 | 0.555 | 0.658 | 3.81E-16 | B-cell_0 |
| LRMP | 2.35E-20 | -0.29417898 | 0.433 | 0.563 | 4.12E-16 | B-cell_0 |
| RNF213 | 3.65E-20 | -0.29788351 | 0.492 | 0.608 | 6.40E-16 | B-cell_0 |
| PARP15 | 4.03E-20 | -0.28359313 | 0.399 | 0.524 | 7.08E-16 | B-cell_0 |
| HDAC9 | 5.58E-20 | -0.26000228 | 0.4 | 0.529 | 9.80E-16 | B-cell_0 |
| DNAJA1 | 1.38E-19 | -0.27944604 | 0.429 | 0.549 | 2.42E-15 | B-cell_0 |
| ACTR2 | 1.74E-19 | -0.26097893 | 0.753 | 0.822 | 3.06E-15 | B-cell_0 |
| CAPG | 2.73E-19 | 0.255332972 | 0.76 | 0.661 | 4.79E-15 | B-cell_0 |
| CCDC88A | 2.74E-19 | 0.272271857 | 0.57 | 0.415 | 4.81E-15 | B-cell_0 |
| ZC3H6 | 3.57E-19 | 0.257496502 | 0.381 | 0.247 | 6.27E-15 | B-cell_0 |
| ID3 | 4.78E-19 | 0.346356164 | 0.533 | 0.389 | 8.39E-15 | B-cell_0 |
| S100A10 | 6.67E-19 | 0.300614047 | 0.866 | 0.794 | 1.17E-14 | B-cell_0 |
| SEC61B | 1.71E-18 | -0.27363278 | 0.397 | 0.511 | 3.01E-14 | B-cell_0 |
| NCL | 2.96E-18 | 0.262193515 | 0.951 | 0.908 | 5.20E-14 | B-cell_0 |
| IGLC1 | 3.08E-18 | -0.33310574 | 0.101 | 0.195 | 5.41E-14 | B-cell_0 |
| ARL4C | 3.52E-18 | -0.3160432 | 0.479 | 0.576 | 6.18E-14 | B-cell_0 |
| LRRFIP1 | 4.58E-17 | -0.25935655 | 0.738 | 0.788 | 8.04E-13 | B-cell_0 |
| SMIM14 | 1.07E-16 | -0.26564407 | 0.77 | 0.827 | 1.88E-12 | B-cell_0 |
| IFT57 | 1.41E-16 | -0.26520698 | 0.542 | 0.627 | 2.48E-12 | B-cell_0 |
| ACAP2 | 4.02E-16 | -0.25342869 | 0.475 | 0.572 | 7.05E-12 | B-cell_0 |
| ALOX5 | 4.08E-16 | -0.25546709 | 0.704 | 0.769 | 7.17E-12 | B-cell_0 |
| KIAA0040 | 1.38E-15 | -0.27676617 | 0.521 | 0.611 | 2.42E-11 | B-cell_0 |
| ZNF148 | 3.65E-15 | -0.26143292 | 0.377 | 0.478 | 6.40E-11 | B-cell_0 |
| SYNE2 | 6.55E-15 | 0.265461434 | 0.836 | 0.733 | 1.15E-10 | B-cell_0 |
| STX7 | 1.78E-14 | -0.27324132 | 0.527 | 0.61 | 3.13E-10 | B-cell_0 |
| ATF7IP | 5.99E-13 | -0.25644276 | 0.606 | 0.669 | 1.05E-08 | B-cell_0 |
| GOLGB1 | 3.04E-12 | -0.29228962 | 0.508 | 0.583 | 5.33E-08 | B-cell_0 |
| FOSB | 2.17E-11 | 0.352924078 | 0.369 | 0.272 | 3.81E-07 | B-cell_0 |
| OTUD1 | 2.70E-11 | -0.25425584 | 0.4 | 0.487 | 4.74E-07 | B-cell_0 |
| MARCKS | 7.25E-10 | -0.27414303 | 0.791 | 0.805 | 1.27E-05 | B-cell_0 |
| EEF1A1.1 | 0 | 0.83675449 | 1 | 1 | 0 | B-cell_1 |
| RPL35.1 | 1.69E-279 | 0.774304424 | 1 | 1 | 2.97E-275 | B-cell_1 |
| ITGA4.1 | 4.71E-263 | 0.669887066 | 0.415 | 0.024 | 8.28E-259 | B-cell_1 |
| MS4A1.1 | 8.43E-243 | 0.953658203 | 0.999 | 0.972 | 1.48E-238 | B-cell_1 |
| SERPINF1.1 | 6.18E-242 | -1.08424602 | 0.063 | 0.652 | 1.09E-237 | B-cell_1 |
| CD69.1 | 9.20E-239 | 1.194097201 | 0.712 | 0.227 | 1.61E-234 | B-cell_1 |
| RPL7A.1 | 1.50E-237 | 0.734070852 | 1 | 0.998 | 2.64E-233 | B-cell_1 |
| TTN.1 | 3.17E-206 | -1.43267839 | 0.198 | 0.72 | 5.57E-202 | B-cell_1 |
| CYBB.1 | 5.64E-193 | 0.756129559 | 0.569 | 0.137 | 9.91E-189 | B-cell_1 |
| RPL12.1 | 9.70E-191 | 0.662215881 | 1 | 0.997 | 1.70E-186 | B-cell_1 |
| APOBEC3H.1 | 2.96E-176 | -0.92031979 | 0.028 | 0.509 | 5.20E-172 | B-cell_1 |
| SET.1 | 5.01E-176 | 0.830742827 | 0.963 | 0.783 | 8.81E-172 | B-cell_1 |
| RRBP1.1 | 1.66E-166 | -1.05426356 | 0.313 | 0.741 | 2.92E-162 | B-cell_1 |
| FCRL1.1 | 1.83E-144 | 0.76604272 | 0.628 | 0.255 | 3.21E-140 | B-cell_1 |
| RPLP1.1 | 2.29E-143 | -0.43943712 | 1 | 1 | 4.02E-139 | B-cell_1 |
| IGHM.1 | 1.12E-141 | -0.68325749 | 0.997 | 0.996 | 1.97E-137 | B-cell_1 |
| NEFL.1 | 6.84E-136 | -0.82931857 | 0.306 | 0.72 | 1.20E-131 | B-cell_1 |
| RPS27A.1 | 5.39E-133 | -0.3915559 | 1 | 1 | 9.46E-129 | B-cell_1 |
| RPS11.1 | 6.33E-133 | 0.449681535 | 0.999 | 0.999 | 1.11E-128 | B-cell_1 |
| B2M.1 | 1.43E-130 | -0.41784124 | 1 | 1 | 2.52E-126 | B-cell_1 |
| PMEPA1.1 | 1.08E-125 | 0.319918217 | 0.176 | 0.003 | 1.90E-121 | B-cell_1 |
| FCRL2.1 | 3.42E-125 | 0.758717888 | 0.743 | 0.414 | 6.01E-121 | B-cell_1 |
| SYK.1 | 7.13E-124 | 0.643311887 | 0.518 | 0.185 | 1.25E-119 | B-cell_1 |
| CDKN2A.1 | 1.57E-123 | 0.297420822 | 0.181 | 0.005 | 2.76E-119 | B-cell_1 |
| RPS6.1 | 2.49E-122 | 0.510105969 | 1 | 1 | 4.37E-118 | B-cell_1 |
| AMN.1 | 1.61E-120 | -0.65656151 | 0.112 | 0.5 | 2.83E-116 | B-cell_1 |
| JCHAIN.1 | 1.80E-115 | -1.32688866 | 0.045 | 0.406 | 3.16E-111 | B-cell_1 |
| CCDC141.1 | 1.62E-113 | -0.67899784 | 0.066 | 0.433 | 2.85E-109 | B-cell_1 |
| AIM2.1 | 3.58E-113 | -0.733552 | 0.455 | 0.776 | 6.28E-109 | B-cell_1 |
| AIRE.1 | 3.07E-111 | -0.79308072 | 0.012 | 0.352 | 5.39E-107 | B-cell_1 |
| RPS7.1 | 2.67E-103 | -0.40865619 | 1 | 1 | 4.69E-99 | B-cell_1 |
| LTB.1 | 1.41E-102 | -0.60733211 | 0.987 | 0.991 | 2.48E-98 | B-cell_1 |
| TSHZ2.1 | 1.65E-102 | -0.67065724 | 0.469 | 0.808 | 2.89E-98 | B-cell_1 |
| MS4A7.1 | 1.07E-100 | 0.349626995 | 0.236 | 0.034 | 1.88E-96 | B-cell_1 |
| RPL41.1 | 1.23E-99 | -0.32315881 | 1 | 1 | 2.16E-95 | B-cell_1 |
| RPL23A.1 | 1.43E-97 | -0.33954834 | 1 | 1 | 2.51E-93 | B-cell_1 |
| MCTP2.1 | 5.16E-96 | 0.522618661 | 0.498 | 0.2 | 9.07E-92 | B-cell_1 |
| RPS21.1 | 6.90E-96 | -0.32342971 | 1 | 1 | 1.21E-91 | B-cell_1 |
| FCMR.1 | 3.86E-93 | 0.575441059 | 0.969 | 0.868 | 6.77E-89 | B-cell_1 |
| RPL21.1 | 1.09E-92 | 0.514212688 | 1 | 1 | 1.92E-88 | B-cell_1 |
| SOX11.1 | 1.78E-91 | 0.506853467 | 0.374 | 0.113 | 3.12E-87 | B-cell_1 |
| TXNIP.1 | 1.86E-88 | -0.58831096 | 0.966 | 0.991 | 3.26E-84 | B-cell_1 |
| WNT3.1 | 5.95E-88 | -0.68342726 | 0.224 | 0.553 | 1.04E-83 | B-cell_1 |
| RPL27A.1 | 2.32E-87 | -0.29254311 | 1 | 1 | 4.07E-83 | B-cell_1 |
| RPS24.1 | 5.18E-87 | -0.31908001 | 1 | 1 | 9.10E-83 | B-cell_1 |
| SRP14.1 | 6.09E-86 | 0.519325696 | 0.93 | 0.846 | 1.07E-81 | B-cell_1 |
| FNBP1.1 | 1.22E-84 | 0.61531574 | 0.763 | 0.533 | 2.14E-80 | B-cell_1 |
| GADD45B.1 | 4.31E-84 | -0.73784703 | 0.389 | 0.682 | 7.57E-80 | B-cell_1 |
| HRK.1 | 4.61E-84 | -0.58276661 | 0.143 | 0.473 | 8.09E-80 | B-cell_1 |
| BMPR2.1 | 3.01E-83 | 0.276649321 | 0.191 | 0.026 | 5.29E-79 | B-cell_1 |
| HCST.1 | 2.46E-82 | -0.56224361 | 0.087 | 0.39 | 4.31E-78 | B-cell_1 |
| EBLN3P.1 | 3.32E-81 | 0.612140228 | 0.735 | 0.509 | 5.82E-77 | B-cell_1 |
| CXCR4.1 | 3.53E-81 | 0.879871779 | 0.755 | 0.557 | 6.21E-77 | B-cell_1 |
| CD74.1 | 1.84E-80 | -0.31548143 | 1 | 1 | 3.23E-76 | B-cell_1 |
| RPL24.1 | 5.95E-79 | 0.400105195 | 1 | 1 | 1.05E-74 | B-cell_1 |
| RPL19.1 | 3.80E-76 | -0.28239799 | 1 | 1 | 6.67E-72 | B-cell_1 |
| S100A4.1 | 4.21E-75 | -0.59108467 | 0.946 | 0.98 | 7.39E-71 | B-cell_1 |
| AC133644.2.1 | 2.07E-74 | -0.44550665 | 0.016 | 0.266 | 3.64E-70 | B-cell_1 |
| KLF13.1 | 2.30E-73 | 0.526456632 | 0.579 | 0.323 | 4.03E-69 | B-cell_1 |
| IGHG3 | 5.56E-70 | -0.7315578 | 0.449 | 0.72 | 9.76E-66 | B-cell_1 |
| APRT.1 | 6.33E-70 | -0.50122711 | 0.558 | 0.789 | 1.11E-65 | B-cell_1 |
| LGALS3.1 | 3.55E-69 | -0.51847041 | 0.179 | 0.463 | 6.24E-65 | B-cell_1 |
| ZBTB20.1 | 1.11E-68 | 0.58208423 | 0.795 | 0.617 | 1.95E-64 | B-cell_1 |
| BTG1.1 | 1.22E-68 | 0.469180482 | 0.977 | 0.942 | 2.13E-64 | B-cell_1 |
| EBF1.1 | 5.98E-68 | 0.484559461 | 0.519 | 0.266 | 1.05E-63 | B-cell_1 |
| RALGPS2.1 | 1.45E-67 | 0.458480376 | 0.986 | 0.942 | 2.55E-63 | B-cell_1 |
| AC253572.2.1 | 2.47E-67 | 0.794051265 | 0.67 | 0.431 | 4.34E-63 | B-cell_1 |
| TOMM7.1 | 2.88E-67 | -0.38401505 | 0.994 | 0.996 | 5.06E-63 | B-cell_1 |
| PAX5.1 | 7.47E-67 | 0.523516171 | 0.868 | 0.75 | 1.31E-62 | B-cell_1 |
| CNTRL.1 | 1.15E-66 | 0.501517527 | 0.599 | 0.343 | 2.02E-62 | B-cell_1 |
| CCDC50.1 | 9.73E-66 | 0.447611508 | 0.94 | 0.858 | 1.71E-61 | B-cell_1 |
| HERC2.1 | 3.49E-65 | 0.518590257 | 0.597 | 0.355 | 6.13E-61 | B-cell_1 |
| HLA-B.1 | 5.99E-65 | -0.27111778 | 1 | 1 | 1.05E-60 | B-cell_1 |
| PCDH9.1 | 7.22E-65 | -0.50296014 | 0.043 | 0.284 | 1.27E-60 | B-cell_1 |
| PABPC1.1 | 1.51E-64 | 0.371782809 | 0.997 | 0.99 | 2.64E-60 | B-cell_1 |
| TMSB4X.1 | 6.11E-64 | -0.33949099 | 1 | 1 | 1.07E-59 | B-cell_1 |
| EPCAM.1 | 1.46E-63 | -0.45185109 | 0.203 | 0.486 | 2.57E-59 | B-cell_1 |
| IGLC3.1 | 3.33E-63 | -0.3259805 | 1 | 0.995 | 5.85E-59 | B-cell_1 |
| SETX.1 | 4.26E-63 | 0.561055636 | 0.611 | 0.378 | 7.48E-59 | B-cell_1 |
| IQSEC1.1 | 9.42E-63 | 0.461199244 | 0.455 | 0.217 | 1.65E-58 | B-cell_1 |
| SERF2.1 | 4.16E-62 | -0.36032339 | 0.991 | 0.998 | 7.30E-58 | B-cell_1 |
| CCDC151.1 | 1.04E-61 | -0.4642787 | 0.198 | 0.481 | 1.82E-57 | B-cell_1 |
| SOD1.1 | 2.80E-61 | -0.42930449 | 0.784 | 0.912 | 4.92E-57 | B-cell_1 |
| ZNF804A.1 | 2.86E-61 | -0.60237288 | 0.293 | 0.555 | 5.03E-57 | B-cell_1 |
| HNRNPK.1 | 5.76E-60 | 0.478690266 | 0.802 | 0.66 | 1.01E-55 | B-cell_1 |
| P2RX5.1 | 1.30E-59 | 0.508431606 | 0.605 | 0.375 | 2.29E-55 | B-cell_1 |
| HLA-A.1 | 1.64E-59 | -0.31490196 | 1 | 1 | 2.88E-55 | B-cell_1 |
| SERPINB6.1 | 2.74E-58 | -0.38639851 | 0.072 | 0.307 | 4.81E-54 | B-cell_1 |
| MEF2A.1 | 4.32E-57 | 0.425170613 | 0.498 | 0.266 | 7.59E-53 | B-cell_1 |
| HNRNPU.1 | 1.38E-56 | 0.481285607 | 0.925 | 0.849 | 2.43E-52 | B-cell_1 |
| MT-CO1.1 | 1.43E-56 | -0.28733775 | 1 | 0.999 | 2.51E-52 | B-cell_1 |
| MPHOSPH8.1 | 2.33E-56 | 0.586597225 | 0.62 | 0.42 | 4.10E-52 | B-cell_1 |
| BTLA.1 | 1.21E-55 | 0.253229214 | 0.18 | 0.04 | 2.13E-51 | B-cell_1 |
| TCF7.1 | 1.23E-54 | -0.45701809 | 0.22 | 0.477 | 2.17E-50 | B-cell_1 |
| TNFRSF1B.1 | 3.36E-54 | -0.39518394 | 0.089 | 0.32 | 5.91E-50 | B-cell_1 |
| STMN1.1 | 3.85E-54 | 0.451676426 | 0.402 | 0.188 | 6.75E-50 | B-cell_1 |
| APOD.1 | 4.78E-54 | -0.46168364 | 0.081 | 0.31 | 8.39E-50 | B-cell_1 |
| UBE3A.1 | 6.94E-54 | 0.397130385 | 0.436 | 0.214 | 1.22E-49 | B-cell_1 |
| EIF1.1 | 2.02E-53 | -0.2719968 | 1 | 1 | 3.54E-49 | B-cell_1 |
| RPL35A.1 | 6.21E-53 | 0.295044784 | 1 | 1 | 1.09E-48 | B-cell_1 |
| RPL10.1 | 8.77E-53 | -0.2541631 | 1 | 1 | 1.54E-48 | B-cell_1 |
| NIBAN3.1 | 1.71E-52 | 0.455251097 | 0.874 | 0.754 | 3.01E-48 | B-cell_1 |
| COX7A2.1 | 2.83E-52 | 0.435730729 | 0.688 | 0.501 | 4.98E-48 | B-cell_1 |
| CBLB.1 | 3.12E-52 | 0.366986032 | 0.421 | 0.203 | 5.47E-48 | B-cell_1 |
| GNG7.1 | 3.42E-52 | -0.35505386 | 0.105 | 0.343 | 6.01E-48 | B-cell_1 |
| FAM214A.1 | 8.89E-52 | 0.371838407 | 0.409 | 0.196 | 1.56E-47 | B-cell_1 |
| SSBP2.1 | 2.17E-51 | -0.32049084 | 0.039 | 0.239 | 3.82E-47 | B-cell_1 |
| SP100.1 | 3.27E-51 | 0.423943409 | 0.965 | 0.91 | 5.75E-47 | B-cell_1 |
| SMPD3.1 | 6.79E-51 | -0.42714958 | 0.171 | 0.404 | 1.19E-46 | B-cell_1 |
| EPB41.1 | 1.34E-50 | -0.44336013 | 0.764 | 0.883 | 2.35E-46 | B-cell_1 |
| GOLGA8B.1 | 6.65E-50 | 0.415396287 | 0.487 | 0.275 | 1.17E-45 | B-cell_1 |
| N4BP2L2.1 | 8.53E-50 | 0.43871059 | 0.783 | 0.632 | 1.50E-45 | B-cell_1 |
| NSMCE3.1 | 4.32E-49 | 0.390365731 | 0.498 | 0.281 | 7.58E-45 | B-cell_1 |
| CD24.1 | 1.09E-48 | -0.46690576 | 0.437 | 0.664 | 1.91E-44 | B-cell_1 |
| HMCES.1 | 2.56E-48 | 0.27888043 | 0.256 | 0.09 | 4.49E-44 | B-cell_1 |
| FOXP1.1 | 5.09E-48 | 0.405091896 | 0.965 | 0.925 | 8.94E-44 | B-cell_1 |
| RPL14.1 | 6.24E-48 | -0.2691265 | 1 | 1 | 1.10E-43 | B-cell_1 |
| LINC00926.1 | 9.18E-48 | 0.442671998 | 0.87 | 0.788 | 1.61E-43 | B-cell_1 |
| APPL1.1 | 1.06E-47 | -0.4221204 | 0.612 | 0.809 | 1.86E-43 | B-cell_1 |
| RSL24D1.1 | 3.92E-47 | 0.415491618 | 0.55 | 0.346 | 6.88E-43 | B-cell_1 |
| LSM10.1 | 7.23E-47 | -0.40630742 | 0.346 | 0.57 | 1.27E-42 | B-cell_1 |
| PSIP1.1 | 8.00E-47 | 0.44590541 | 0.621 | 0.422 | 1.40E-42 | B-cell_1 |
| HLA-C.1 | 8.58E-47 | -0.2985846 | 0.996 | 0.998 | 1.51E-42 | B-cell_1 |
| ATP6V1G1.1 | 1.41E-46 | 0.398755194 | 0.733 | 0.563 | 2.48E-42 | B-cell_1 |
| CRIP2.1 | 4.28E-46 | -0.43192606 | 0.415 | 0.653 | 7.52E-42 | B-cell_1 |
| RAPGEF1.1 | 1.65E-45 | 0.341390091 | 0.407 | 0.206 | 2.89E-41 | B-cell_1 |
| SHISA8.1 | 2.29E-45 | -0.31076365 | 0.046 | 0.232 | 4.03E-41 | B-cell_1 |
| SHTN1.1 | 2.96E-45 | -0.27575339 | 0.016 | 0.185 | 5.20E-41 | B-cell_1 |
| ACSM3.1 | 7.97E-45 | 0.314412963 | 0.248 | 0.092 | 1.40E-40 | B-cell_1 |
| AC245014.3.1 | 1.22E-44 | 0.494717974 | 0.369 | 0.178 | 2.14E-40 | B-cell_1 |
| PARP14.1 | 2.24E-44 | 0.490190946 | 0.574 | 0.379 | 3.93E-40 | B-cell_1 |
| B4GALT1.1 | 3.13E-44 | 0.374749829 | 0.453 | 0.252 | 5.49E-40 | B-cell_1 |
| AC103591.3.1 | 3.48E-44 | 0.517899682 | 0.332 | 0.151 | 6.11E-40 | B-cell_1 |
| PTPRC.1 | 7.93E-44 | 0.34148927 | 0.984 | 0.969 | 1.39E-39 | B-cell_1 |
| CD27.1 | 9.42E-44 | -0.41182817 | 0.41 | 0.628 | 1.65E-39 | B-cell_1 |
| CRIP1.1 | 1.39E-43 | -0.43749207 | 0.965 | 0.989 | 2.44E-39 | B-cell_1 |
| DSP.1 | 2.16E-43 | 0.290767511 | 0.162 | 0.041 | 3.80E-39 | B-cell_1 |
| UGCG.1 | 2.19E-43 | 0.35885951 | 0.411 | 0.217 | 3.85E-39 | B-cell_1 |
| TUT7.1 | 3.89E-43 | 0.33866075 | 0.345 | 0.162 | 6.82E-39 | B-cell_1 |
| ZMYM2.1 | 5.05E-43 | 0.349523801 | 0.371 | 0.184 | 8.87E-39 | B-cell_1 |
| SNX22.1 | 4.11E-42 | 0.256758657 | 0.234 | 0.083 | 7.22E-38 | B-cell_1 |
| ITPR2.1 | 4.54E-42 | 0.454221755 | 0.563 | 0.368 | 7.97E-38 | B-cell_1 |
| GNB5.1 | 5.22E-42 | 0.322033627 | 0.343 | 0.163 | 9.17E-38 | B-cell_1 |
| GAPT.1 | 2.31E-41 | 0.321546744 | 0.319 | 0.144 | 4.06E-37 | B-cell_1 |
| FXYD1 | 1.05E-40 | -0.26565132 | 0.021 | 0.178 | 1.84E-36 | B-cell_1 |
| UBE2R2.1 | 1.83E-40 | 0.326728318 | 0.397 | 0.21 | 3.21E-36 | B-cell_1 |
| SERP1.1 | 2.62E-40 | 0.372968197 | 0.838 | 0.739 | 4.60E-36 | B-cell_1 |
| SAV1.1 | 4.01E-40 | 0.275307219 | 0.275 | 0.114 | 7.04E-36 | B-cell_1 |
| ROMO1.1 | 1.39E-39 | -0.3672338 | 0.515 | 0.71 | 2.44E-35 | B-cell_1 |
| TUT4.1 | 5.24E-39 | 0.413424747 | 0.766 | 0.653 | 9.19E-35 | B-cell_1 |
| IFNG-AS1.1 | 7.55E-39 | -0.32677773 | 0.185 | 0.398 | 1.32E-34 | B-cell_1 |
| APOBEC3G.1 | 1.03E-38 | -0.38463581 | 0.389 | 0.592 | 1.81E-34 | B-cell_1 |
| RAD23B.1 | 1.34E-38 | 0.293611535 | 0.365 | 0.187 | 2.36E-34 | B-cell_1 |
| TRA2B.1 | 1.41E-38 | 0.295461485 | 0.989 | 0.956 | 2.47E-34 | B-cell_1 |
| H1FX.1 | 2.12E-38 | 0.502090502 | 0.934 | 0.812 | 3.73E-34 | B-cell_1 |
| PPIB.1 | 8.05E-38 | -0.36074551 | 0.65 | 0.818 | 1.41E-33 | B-cell_1 |
| TCL1A.1 | 8.33E-38 | 0.433922657 | 0.548 | 0.359 | 1.46E-33 | B-cell_1 |
| SQSTM1.1 | 9.22E-38 | -0.40293657 | 0.483 | 0.675 | 1.62E-33 | B-cell_1 |
| TXNDC5 | 9.57E-38 | -0.35085165 | 0.146 | 0.343 | 1.68E-33 | B-cell_1 |
| SECISBP2.1 | 1.40E-37 | 0.338992508 | 0.442 | 0.257 | 2.46E-33 | B-cell_1 |
| ZCCHC7.1 | 3.15E-37 | 0.389022565 | 0.545 | 0.36 | 5.53E-33 | B-cell_1 |
| ELOVL5.1 | 3.95E-37 | -0.34854167 | 0.478 | 0.674 | 6.94E-33 | B-cell_1 |
| HSP90B1.1 | 4.52E-37 | -0.48265756 | 0.603 | 0.77 | 7.94E-33 | B-cell_1 |
| GANC.1 | 4.63E-37 | 0.256280083 | 0.271 | 0.117 | 8.13E-33 | B-cell_1 |
| RTF1.1 | 6.19E-37 | 0.356389541 | 0.494 | 0.303 | 1.09E-32 | B-cell_1 |
| CALR.1 | 9.37E-37 | -0.46578819 | 0.567 | 0.732 | 1.64E-32 | B-cell_1 |
| RYR1.1 | 1.53E-36 | -0.2616075 | 0.061 | 0.227 | 2.68E-32 | B-cell_1 |
| AKAP13.1 | 3.06E-36 | 0.318668668 | 0.946 | 0.888 | 5.38E-32 | B-cell_1 |
| CEP170.1 | 3.73E-36 | 0.294283112 | 0.298 | 0.139 | 6.56E-32 | B-cell_1 |
| GOLGA8A.1 | 3.84E-36 | 0.339617167 | 0.461 | 0.278 | 6.74E-32 | B-cell_1 |
| PTBP3.1 | 5.14E-36 | 0.379905055 | 0.492 | 0.317 | 9.03E-32 | B-cell_1 |
| ACTB | 8.14E-36 | -0.25861497 | 1 | 1 | 1.43E-31 | B-cell_1 |
| ICAM3.1 | 9.97E-36 | -0.34060952 | 0.508 | 0.692 | 1.75E-31 | B-cell_1 |
| ITGB1.1 | 1.55E-35 | -0.38213132 | 0.074 | 0.24 | 2.72E-31 | B-cell_1 |
| STRBP.1 | 2.13E-35 | 0.394903803 | 0.609 | 0.444 | 3.75E-31 | B-cell_1 |
| CUTA.1 | 5.14E-35 | -0.32585245 | 0.455 | 0.654 | 9.02E-31 | B-cell_1 |
| SKIL.1 | 9.80E-35 | 0.39170864 | 0.45 | 0.275 | 1.72E-30 | B-cell_1 |
| SAP18.1 | 9.88E-35 | 0.371410442 | 0.583 | 0.42 | 1.74E-30 | B-cell_1 |
| HMGB1.1 | 1.47E-34 | 0.34951439 | 0.942 | 0.914 | 2.58E-30 | B-cell_1 |
| RASSF6.1 | 1.80E-34 | -0.46248469 | 0.119 | 0.297 | 3.16E-30 | B-cell_1 |
| ETS1.1 | 2.56E-34 | 0.398389148 | 0.799 | 0.699 | 4.49E-30 | B-cell_1 |
| SSBP3.1 | 5.95E-34 | -0.31524454 | 0.21 | 0.409 | 1.04E-29 | B-cell_1 |
| ELK4.1 | 7.46E-34 | 0.348523653 | 0.415 | 0.248 | 1.31E-29 | B-cell_1 |
| PNRC1.1 | 9.63E-34 | 0.394394073 | 0.887 | 0.831 | 1.69E-29 | B-cell_1 |
| SEC62 | 2.10E-33 | 0.290066696 | 0.945 | 0.903 | 3.69E-29 | B-cell_1 |
| HCK.1 | 2.43E-33 | 0.28816269 | 0.347 | 0.182 | 4.27E-29 | B-cell_1 |
| RESF1.1 | 2.45E-33 | 0.408090285 | 0.858 | 0.783 | 4.30E-29 | B-cell_1 |
| POU2F2.1 | 3.89E-33 | 0.400012516 | 0.856 | 0.78 | 6.83E-29 | B-cell_1 |
| PRDX1.1 | 6.42E-33 | -0.3324703 | 0.552 | 0.724 | 1.13E-28 | B-cell_1 |
| ANP32B.1 | 6.68E-33 | 0.358912047 | 0.713 | 0.581 | 1.17E-28 | B-cell_1 |
| KIAA2026.1 | 7.78E-33 | 0.323034129 | 0.444 | 0.267 | 1.37E-28 | B-cell_1 |
| ATP5F1E | 9.65E-33 | -0.25157731 | 0.992 | 0.999 | 1.69E-28 | B-cell_1 |
| ACP5.1 | 1.11E-32 | -0.36897366 | 0.448 | 0.632 | 1.96E-28 | B-cell_1 |
| OIP5-AS1.1 | 1.72E-32 | 0.333134681 | 0.452 | 0.286 | 3.02E-28 | B-cell_1 |
| TTF1.1 | 3.07E-32 | 0.252034282 | 0.25 | 0.11 | 5.39E-28 | B-cell_1 |
| SMCHD1.1 | 4.64E-32 | 0.313233989 | 0.945 | 0.905 | 8.15E-28 | B-cell_1 |
| NME2.1 | 4.86E-32 | -0.30361531 | 0.942 | 0.976 | 8.54E-28 | B-cell_1 |
| SENP6.1 | 4.88E-32 | 0.30705189 | 0.426 | 0.254 | 8.56E-28 | B-cell_1 |
| KATNBL1.1 | 5.60E-32 | 0.270362726 | 0.329 | 0.172 | 9.84E-28 | B-cell_1 |
| PLEKHA2.1 | 6.15E-32 | 0.35681612 | 0.767 | 0.65 | 1.08E-27 | B-cell_1 |
| ATRX.1 | 7.75E-32 | 0.354832124 | 0.89 | 0.828 | 1.36E-27 | B-cell_1 |
| PALM2-AKAP2.1 | 8.56E-32 | 0.344693641 | 0.434 | 0.265 | 1.50E-27 | B-cell_1 |
| CKLF.1 | 1.19E-31 | -0.31877945 | 0.267 | 0.455 | 2.09E-27 | B-cell_1 |
| OSTF1.1 | 1.61E-31 | 0.278099845 | 0.347 | 0.19 | 2.82E-27 | B-cell_1 |
| CAMK2D.1 | 1.94E-31 | 0.327752623 | 0.397 | 0.233 | 3.40E-27 | B-cell_1 |
| BCL11A.1 | 3.47E-31 | 0.33785111 | 0.874 | 0.802 | 6.09E-27 | B-cell_1 |
| ZNF106.1 | 3.67E-31 | 0.301190431 | 0.359 | 0.2 | 6.44E-27 | B-cell_1 |
| PHPT1.1 | 1.05E-30 | 0.251003432 | 0.286 | 0.139 | 1.84E-26 | B-cell_1 |
| SIAH2.1 | 1.22E-30 | 0.293036698 | 0.442 | 0.274 | 2.14E-26 | B-cell_1 |
| ARPC1B.1 | 1.44E-30 | -0.32805025 | 0.806 | 0.892 | 2.54E-26 | B-cell_1 |
| MAP1LC3A.1 | 1.69E-30 | -0.25653909 | 0.049 | 0.189 | 2.97E-26 | B-cell_1 |
| PRRC2B.1 | 1.96E-30 | 0.302002571 | 0.433 | 0.266 | 3.44E-26 | B-cell_1 |
| FAM120AOS.1 | 2.53E-30 | 0.289667965 | 0.379 | 0.217 | 4.45E-26 | B-cell_1 |
| CIRBP.1 | 2.99E-30 | -0.2805292 | 0.914 | 0.963 | 5.25E-26 | B-cell_1 |
| GPSM3.1 | 3.20E-30 | 0.339263975 | 0.642 | 0.501 | 5.63E-26 | B-cell_1 |
| CD47.1 | 3.83E-30 | 0.361772386 | 0.682 | 0.554 | 6.72E-26 | B-cell_1 |
| TLN1.1 | 4.16E-30 | 0.360901824 | 0.59 | 0.445 | 7.30E-26 | B-cell_1 |
| EDF1.1 | 4.21E-30 | 0.318801816 | 0.755 | 0.653 | 7.39E-26 | B-cell_1 |
| C9orf78.1 | 1.57E-29 | 0.323626913 | 0.473 | 0.318 | 2.76E-25 | B-cell_1 |
| UHRF2 | 1.91E-29 | 0.260134158 | 0.332 | 0.179 | 3.36E-25 | B-cell_1 |
| VAMP8.1 | 3.80E-29 | -0.28861943 | 0.505 | 0.69 | 6.66E-25 | B-cell_1 |
| PLEKHG1 | 6.40E-29 | 0.25614521 | 0.351 | 0.194 | 1.12E-24 | B-cell_1 |
| CDV3.1 | 6.52E-29 | 0.301024168 | 0.868 | 0.808 | 1.15E-24 | B-cell_1 |
| ATP5MPL.1 | 7.81E-29 | -0.28668933 | 0.604 | 0.771 | 1.37E-24 | B-cell_1 |
| RPL22L1.1 | 3.81E-28 | 0.327158928 | 0.494 | 0.336 | 6.68E-24 | B-cell_1 |
| ZNF107.1 | 4.33E-28 | 0.345702229 | 0.493 | 0.336 | 7.61E-24 | B-cell_1 |
| SMC5.1 | 4.34E-28 | 0.268899404 | 0.332 | 0.184 | 7.61E-24 | B-cell_1 |
| SH3BP5.1 | 4.51E-28 | -0.28458133 | 0.772 | 0.879 | 7.92E-24 | B-cell_1 |
| CORO1B.1 | 5.33E-28 | -0.322024 | 0.448 | 0.622 | 9.37E-24 | B-cell_1 |
| GTF2I.1 | 8.61E-28 | 0.319857646 | 0.768 | 0.651 | 1.51E-23 | B-cell_1 |
| CENPM.1 | 1.50E-27 | -0.29056793 | 0.229 | 0.403 | 2.63E-23 | B-cell_1 |
| SMC6.1 | 1.59E-27 | 0.34565154 | 0.519 | 0.366 | 2.79E-23 | B-cell_1 |
| PDIA3.1 | 1.76E-27 | -0.30755483 | 0.468 | 0.647 | 3.09E-23 | B-cell_1 |
| FAM107B.1 | 1.85E-27 | 0.379846892 | 0.678 | 0.566 | 3.26E-23 | B-cell_1 |
| CNTNAP2.1 | 2.84E-27 | -0.25710251 | 0.09 | 0.239 | 4.98E-23 | B-cell_1 |
| SENP7.1 | 3.88E-27 | 0.310896727 | 0.39 | 0.241 | 6.82E-23 | B-cell_1 |
| HLA-DRB1 | 5.12E-27 | -0.33604753 | 0.962 | 0.982 | 9.00E-23 | B-cell_1 |
| ZFAND5.1 | 5.37E-27 | 0.260290724 | 0.391 | 0.234 | 9.43E-23 | B-cell_1 |
| WASHC4.1 | 6.60E-27 | 0.317113589 | 0.525 | 0.369 | 1.16E-22 | B-cell_1 |
| BMP3.1 | 8.24E-27 | -0.26451917 | 0.118 | 0.271 | 1.45E-22 | B-cell_1 |
| CCND1.1 | 1.45E-26 | -0.41153349 | 0.847 | 0.913 | 2.54E-22 | B-cell_1 |
| JUN.1 | 1.53E-26 | -0.31780302 | 0.984 | 0.992 | 2.69E-22 | B-cell_1 |
| SCIMP.1 | 2.67E-26 | -0.30838576 | 0.439 | 0.617 | 4.68E-22 | B-cell_1 |
| HNRNPA1.1 | 2.99E-26 | 0.290907337 | 0.965 | 0.946 | 5.25E-22 | B-cell_1 |
| VIM | 3.53E-26 | 0.267277316 | 0.992 | 0.985 | 6.20E-22 | B-cell_1 |
| HNRNPDL.1 | 4.90E-26 | 0.294833605 | 0.896 | 0.849 | 8.61E-22 | B-cell_1 |
| COX20.1 | 5.67E-26 | 0.310281425 | 0.509 | 0.36 | 9.95E-22 | B-cell_1 |
| SNHG7.1 | 7.62E-26 | 0.326633423 | 0.59 | 0.447 | 1.34E-21 | B-cell_1 |
| SLTM.1 | 1.27E-25 | 0.368451911 | 0.607 | 0.47 | 2.23E-21 | B-cell_1 |
| C16orf74.1 | 1.60E-25 | -0.27367293 | 0.307 | 0.481 | 2.81E-21 | B-cell_1 |
| EHMT1.1 | 2.11E-25 | 0.30065056 | 0.473 | 0.323 | 3.70E-21 | B-cell_1 |
| TFRC.1 | 2.38E-25 | -0.29673925 | 0.3 | 0.471 | 4.19E-21 | B-cell_1 |
| NCR3.1 | 2.53E-25 | -0.30209052 | 0.365 | 0.536 | 4.44E-21 | B-cell_1 |
| ALOX5.1 | 3.28E-25 | 0.310239524 | 0.797 | 0.696 | 5.77E-21 | B-cell_1 |
| FCRL3.1 | 3.69E-25 | 0.294266083 | 0.422 | 0.27 | 6.47E-21 | B-cell_1 |
| UCP2.1 | 4.76E-25 | -0.28455751 | 0.933 | 0.964 | 8.35E-21 | B-cell_1 |
| DNMBP.1 | 6.38E-25 | -0.25417964 | 0.155 | 0.306 | 1.12E-20 | B-cell_1 |
| UTRN.1 | 1.03E-24 | 0.356695216 | 0.56 | 0.42 | 1.81E-20 | B-cell_1 |
| TRIM22.1 | 1.15E-24 | 0.322005976 | 0.526 | 0.386 | 2.01E-20 | B-cell_1 |
| APOBEC3C.1 | 1.23E-24 | -0.29106349 | 0.403 | 0.567 | 2.15E-20 | B-cell_1 |
| TPM3.1 | 1.67E-24 | 0.299213224 | 0.91 | 0.874 | 2.94E-20 | B-cell_1 |
| MYO1E.1 | 4.39E-24 | -0.28728872 | 0.373 | 0.534 | 7.71E-20 | B-cell_1 |
| FLNB.1 | 5.17E-24 | -0.2515711 | 0.184 | 0.337 | 9.07E-20 | B-cell_1 |
| HDAC9.1 | 5.19E-24 | 0.285310242 | 0.545 | 0.397 | 9.11E-20 | B-cell_1 |
| TRAPPC5.1 | 5.68E-24 | -0.2752176 | 0.637 | 0.788 | 9.97E-20 | B-cell_1 |
| SNHG8.1 | 7.57E-24 | -0.27242664 | 0.604 | 0.745 | 1.33E-19 | B-cell_1 |
| DHX36.1 | 1.09E-23 | 0.307316663 | 0.605 | 0.465 | 1.92E-19 | B-cell_1 |
| SMIM14.1 | 1.56E-23 | 0.316031833 | 0.845 | 0.765 | 2.74E-19 | B-cell_1 |
| ZNF217 | 1.67E-23 | 0.25397882 | 0.373 | 0.23 | 2.94E-19 | B-cell_1 |
| SPTAN1.1 | 2.59E-23 | 0.281557263 | 0.435 | 0.293 | 4.54E-19 | B-cell_1 |
| CDK2AP2.1 | 3.44E-23 | -0.28493735 | 0.232 | 0.383 | 6.05E-19 | B-cell_1 |
| PGGHG | 5.79E-23 | 0.277723916 | 0.454 | 0.31 | 1.02E-18 | B-cell_1 |
| SPTLC2.1 | 8.79E-23 | -0.25283729 | 0.227 | 0.381 | 1.54E-18 | B-cell_1 |
| SLC2A3 | 1.17E-22 | 0.250737257 | 0.285 | 0.157 | 2.05E-18 | B-cell_1 |
| ARL4C.1 | 1.19E-22 | 0.344546629 | 0.594 | 0.475 | 2.08E-18 | B-cell_1 |
| MBD4.1 | 1.41E-22 | 0.316806142 | 0.656 | 0.543 | 2.48E-18 | B-cell_1 |
| DNAJA1.1 | 4.36E-22 | 0.30089713 | 0.559 | 0.428 | 7.66E-18 | B-cell_1 |
| RAB30 | 4.86E-22 | 0.255666495 | 0.435 | 0.291 | 8.53E-18 | B-cell_1 |
| DHRS7.1 | 1.94E-21 | -0.25894114 | 0.417 | 0.569 | 3.40E-17 | B-cell_1 |
| BIN2 | 2.40E-21 | -0.25123317 | 0.229 | 0.378 | 4.21E-17 | B-cell_1 |
| PARP15.1 | 3.95E-21 | 0.294414264 | 0.531 | 0.4 | 6.94E-17 | B-cell_1 |
| CALHM6 | 4.72E-21 | -0.25520706 | 0.357 | 0.514 | 8.29E-17 | B-cell_1 |
| AC044849.1.1 | 5.45E-21 | -0.31535291 | 0.417 | 0.567 | 9.57E-17 | B-cell_1 |
| KIAA0040.1 | 6.72E-21 | 0.315915729 | 0.629 | 0.517 | 1.18E-16 | B-cell_1 |
| NUCKS1.1 | 2.33E-20 | 0.293499616 | 0.665 | 0.555 | 4.10E-16 | B-cell_1 |
| FAM120A | 4.28E-20 | 0.252051664 | 0.413 | 0.281 | 7.52E-16 | B-cell_1 |
| WIPF1.1 | 4.51E-20 | 0.26056477 | 0.458 | 0.324 | 7.91E-16 | B-cell_1 |
| RSRC1 | 7.05E-20 | 0.266651282 | 0.519 | 0.383 | 1.24E-15 | B-cell_1 |
| BTG2 | 1.23E-19 | 0.268243995 | 0.907 | 0.871 | 2.16E-15 | B-cell_1 |
| LRMP.1 | 1.80E-19 | 0.289134591 | 0.566 | 0.435 | 3.17E-15 | B-cell_1 |
| S100A10.1 | 1.87E-19 | -0.31979061 | 0.794 | 0.865 | 3.28E-15 | B-cell_1 |
| POLR1D.1 | 2.27E-19 | 0.254000385 | 0.485 | 0.358 | 3.98E-15 | B-cell_1 |
| LRRFIP1.1 | 2.68E-19 | 0.27469859 | 0.795 | 0.737 | 4.71E-15 | B-cell_1 |
| IFT57.1 | 2.99E-19 | 0.283890936 | 0.64 | 0.539 | 5.25E-15 | B-cell_1 |
| ACTR2.1 | 3.01E-19 | 0.262330726 | 0.823 | 0.754 | 5.29E-15 | B-cell_1 |
| ADI1.1 | 1.06E-18 | -0.25556124 | 0.347 | 0.485 | 1.86E-14 | B-cell_1 |
| BPTF | 2.10E-18 | 0.265216753 | 0.835 | 0.792 | 3.69E-14 | B-cell_1 |
| CHD7 | 5.04E-18 | 0.260321848 | 0.636 | 0.524 | 8.84E-14 | B-cell_1 |
| ZFP36L1 | 5.39E-18 | 0.254718219 | 0.748 | 0.66 | 9.47E-14 | B-cell_1 |
| TUBB4B | 5.95E-18 | 0.25268905 | 0.539 | 0.414 | 1.05E-13 | B-cell_1 |
| MFHAS1 | 9.60E-18 | 0.296892652 | 0.674 | 0.587 | 1.69E-13 | B-cell_1 |
| ZNF148.1 | 1.18E-17 | 0.283832444 | 0.489 | 0.375 | 2.07E-13 | B-cell_1 |
| ATF7IP.1 | 1.27E-17 | 0.295087535 | 0.687 | 0.601 | 2.23E-13 | B-cell_1 |
| BICD2 | 1.33E-17 | 0.250537134 | 0.406 | 0.287 | 2.33E-13 | B-cell_1 |
| ACAP2.1 | 2.37E-17 | 0.2690471 | 0.577 | 0.475 | 4.17E-13 | B-cell_1 |
| RNF213.1 | 2.92E-17 | 0.275740098 | 0.604 | 0.495 | 5.14E-13 | B-cell_1 |
| RHOB.1 | 2.63E-16 | -0.27411291 | 0.596 | 0.71 | 4.61E-12 | B-cell_1 |
| SPTBN1 | 4.66E-16 | 0.264301793 | 0.649 | 0.56 | 8.18E-12 | B-cell_1 |
| PEG10.1 | 1.16E-15 | -0.29593289 | 0.313 | 0.436 | 2.04E-11 | B-cell_1 |
| BDP1 | 1.78E-15 | 0.26165406 | 0.724 | 0.648 | 3.12E-11 | B-cell_1 |
| ID3.1 | 1.88E-15 | -0.31722609 | 0.397 | 0.527 | 3.31E-11 | B-cell_1 |
| TRIM38 | 1.31E-14 | 0.259005166 | 0.635 | 0.542 | 2.30E-10 | B-cell_1 |
| STX7.1 | 3.79E-14 | 0.250530508 | 0.612 | 0.528 | 6.65E-10 | B-cell_1 |
| GOLGB1.1 | 1.12E-13 | 0.303285565 | 0.589 | 0.507 | 1.96E-09 | B-cell_1 |
| MARCKS.1 | 4.93E-13 | 0.302095827 | 0.82 | 0.786 | 8.66E-09 | B-cell_1 |
| FOSB.1 | 3.13E-12 | -0.35687215 | 0.266 | 0.369 | 5.49E-08 | B-cell_1 |
| BBX | 9.92E-12 | 0.26216668 | 0.705 | 0.673 | 1.74E-07 | B-cell_1 |
| TUBA1A | 1.70E-11 | 0.266665469 | 0.771 | 0.731 | 2.99E-07 | B-cell_1 |
| IGLC6 | 8.65E-09 | 0.294106651 | 0.608 | 0.557 | 0.0001519 | B-cell_1 |
| FAM30A | 0 | 0.96685015 | 0.368 | 0.001 | 0 | B-cell_2 |
| HOPX | 5.63E-208 | 0.463897059 | 0.25 | 0.001 | 9.88E-204 | B-cell_2 |
| PAG1 | 3.93E-153 | 0.432870163 | 0.25 | 0.002 | 6.90E-149 | B-cell_2 |
| IGKC | 1.17E-148 | 6.344552111 | 0.706 | 0.04 | 2.05E-144 | B-cell_2 |
| SOX4 | 5.85E-130 | 0.708622226 | 0.309 | 0.006 | 1.03E-125 | B-cell_2 |
| GCSAM | 1.54E-120 | 0.251740793 | 0.147 | 0 | 2.70E-116 | B-cell_2 |
| NIBAN1 | 2.61E-115 | 0.335895532 | 0.191 | 0.002 | 4.58E-111 | B-cell_2 |
| SERPINA1 | 3.34E-89 | 0.541570558 | 0.103 | 0 | 5.86E-85 | B-cell_2 |
| CDK6 | 1.80E-83 | 0.250771782 | 0.132 | 0.001 | 3.17E-79 | B-cell_2 |
| ANXA4 | 4.59E-82 | 0.523904028 | 0.279 | 0.009 | 8.06E-78 | B-cell_2 |
| NKG7 | 3.51E-79 | 0.435452746 | 0.103 | 0 | 6.16E-75 | B-cell_2 |
| ITGAX | 4.20E-72 | 0.626944314 | 0.25 | 0.009 | 7.37E-68 | B-cell_2 |
| SLCO3A1 | 3.79E-71 | 0.250771782 | 0.118 | 0.001 | 6.66E-67 | B-cell_2 |
| SLAMF1 | 6.65E-62 | 0.265892934 | 0.147 | 0.003 | 1.17E-57 | B-cell_2 |
| CCND2 | 2.79E-56 | 0.373156963 | 0.221 | 0.009 | 4.90E-52 | B-cell_2 |
| ITGA4.2 | 3.41E-52 | 1.203855734 | 0.706 | 0.115 | 5.98E-48 | B-cell_2 |
| LMO2 | 6.81E-48 | 0.28846197 | 0.221 | 0.011 | 1.20E-43 | B-cell_2 |
| IGLC3.2 | 3.60E-40 | -1.96307584 | 0.765 | 1 | 6.33E-36 | B-cell_2 |
| THEMIS2 | 3.79E-35 | 0.361369392 | 0.235 | 0.018 | 6.65E-31 | B-cell_2 |
| RASGRP3 | 3.49E-31 | 0.43547337 | 0.235 | 0.021 | 6.13E-27 | B-cell_2 |
| IGHM.2 | 4.01E-31 | -2.10005508 | 0.824 | 0.998 | 7.04E-27 | B-cell_2 |
| FCER1G | 1.61E-30 | 1.005084316 | 0.103 | 0.004 | 2.82E-26 | B-cell_2 |
| RPL12.2 | 5.31E-30 | 1.135360654 | 1 | 0.998 | 9.32E-26 | B-cell_2 |
| CCND1.2 | 5.57E-30 | -2.24866676 | 0.353 | 0.904 | 9.78E-26 | B-cell_2 |
| TEX9 | 9.26E-29 | 0.265442536 | 0.191 | 0.015 | 1.63E-24 | B-cell_2 |
| SCPEP1 | 7.73E-27 | 0.515570721 | 0.279 | 0.034 | 1.36E-22 | B-cell_2 |
| RPS6.2 | 5.25E-26 | 1.022081168 | 1 | 1 | 9.21E-22 | B-cell_2 |
| IGLC7 | 2.71E-25 | -1.85848251 | 0.235 | 0.844 | 4.76E-21 | B-cell_2 |
| RASGRP1 | 1.64E-23 | 0.60289884 | 0.368 | 0.064 | 2.88E-19 | B-cell_2 |
| ITGB2 | 2.09E-23 | 0.961489587 | 0.515 | 0.126 | 3.67E-19 | B-cell_2 |
| EAF2 | 2.24E-23 | 0.766513585 | 0.676 | 0.21 | 3.93E-19 | B-cell_2 |
| TPT1 | 9.14E-23 | 0.920528781 | 1 | 1 | 1.60E-18 | B-cell_2 |
| TBC1D9 | 1.13E-22 | -1.25100131 | 0.191 | 0.8 | 1.98E-18 | B-cell_2 |
| BCL2 | 1.25E-22 | -1.20147751 | 0.353 | 0.899 | 2.19E-18 | B-cell_2 |
| FOXP1.2 | 8.77E-22 | -1.20310016 | 0.5 | 0.942 | 1.54E-17 | B-cell_2 |
| AFF3 | 1.35E-21 | -1.17252721 | 0.5 | 0.925 | 2.37E-17 | B-cell_2 |
| RPS11.2 | 4.12E-21 | 0.673534674 | 1 | 0.999 | 7.23E-17 | B-cell_2 |
| KCNK6 | 1.40E-19 | 0.392165465 | 0.191 | 0.022 | 2.46E-15 | B-cell_2 |
| TSHZ2.2 | 5.42E-19 | -1.22437678 | 0.176 | 0.73 | 9.52E-15 | B-cell_2 |
| KLRK1 | 1.29E-18 | -1.09349003 | 0.088 | 0.667 | 2.27E-14 | B-cell_2 |
| PLD4 | 1.58E-18 | 0.284557676 | 0.221 | 0.03 | 2.78E-14 | B-cell_2 |
| ARPC5L | 1.83E-18 | 0.588341475 | 0.485 | 0.13 | 3.22E-14 | B-cell_2 |
| RPL35.2 | 1.89E-18 | 0.689168319 | 1 | 1 | 3.32E-14 | B-cell_2 |
| H1FX.2 | 1.90E-18 | -1.71739856 | 0.471 | 0.849 | 3.34E-14 | B-cell_2 |
| IGHG1 | 3.51E-18 | 2.368494016 | 0.676 | 0.324 | 6.17E-14 | B-cell_2 |
| RPL37 | 5.94E-18 | 0.633575378 | 1 | 1 | 1.04E-13 | B-cell_2 |
| IFITM2 | 9.76E-18 | 0.924049551 | 0.559 | 0.198 | 1.71E-13 | B-cell_2 |
| NEFL.2 | 2.22E-17 | -1.17864015 | 0.059 | 0.623 | 3.90E-13 | B-cell_2 |
| RPL21.2 | 2.62E-17 | 0.748991285 | 1 | 1 | 4.61E-13 | B-cell_2 |
| HLA-DPB1 | 6.84E-17 | 1.199108164 | 0.897 | 0.942 | 1.20E-12 | B-cell_2 |
| ARHGAP24 | 2.29E-16 | -0.94884169 | 0.368 | 0.827 | 4.02E-12 | B-cell_2 |
| TMEM156 | 2.98E-16 | 0.547578973 | 0.441 | 0.122 | 5.24E-12 | B-cell_2 |
| TCF4 | 3.89E-16 | -1.02594523 | 0.662 | 0.921 | 6.83E-12 | B-cell_2 |
| LYZ | 7.25E-16 | 1.938583477 | 0.103 | 0.008 | 1.27E-11 | B-cell_2 |
| IGLC6.1 | 2.03E-15 | -1.59970928 | 0.059 | 0.578 | 3.56E-11 | B-cell_2 |
| MTSS1.1 | 4.76E-15 | -0.8812869 | 0.662 | 0.934 | 8.36E-11 | B-cell_2 |
| RPL7A.2 | 4.93E-15 | 0.613485557 | 1 | 0.999 | 8.65E-11 | B-cell_2 |
| CCDC50.2 | 5.37E-15 | -0.89498084 | 0.471 | 0.885 | 9.43E-11 | B-cell_2 |
| MFHAS1.1 | 6.87E-15 | -0.9619297 | 0.118 | 0.617 | 1.21E-10 | B-cell_2 |
| SEMA4A | 7.31E-15 | 0.366741499 | 0.176 | 0.024 | 1.28E-10 | B-cell_2 |
| LTB.2 | 7.34E-15 | -0.99937797 | 0.794 | 0.993 | 1.29E-10 | B-cell_2 |
| CD79B | 9.74E-15 | -0.81412671 | 0.838 | 0.988 | 1.71E-10 | B-cell_2 |
| MSI2 | 1.08E-14 | -0.93992976 | 0.162 | 0.629 | 1.90E-10 | B-cell_2 |
| RPS2 | 2.13E-14 | 0.558083106 | 1 | 1 | 3.74E-10 | B-cell_2 |
| LAPTM5 | 3.10E-14 | 1.060646476 | 0.926 | 0.817 | 5.45E-10 | B-cell_2 |
| EPB41.2 | 4.52E-14 | -0.91400754 | 0.574 | 0.857 | 7.93E-10 | B-cell_2 |
| AIF1 | 5.55E-14 | 1.390261235 | 0.147 | 0.019 | 9.74E-10 | B-cell_2 |
| LILRB2 | 6.57E-14 | 0.264169756 | 0.132 | 0.015 | 1.15E-09 | B-cell_2 |
| S100A4.2 | 7.00E-14 | -0.74717483 | 0.809 | 0.974 | 1.23E-09 | B-cell_2 |
| ALOX5.2 | 7.63E-14 | -0.85641746 | 0.294 | 0.728 | 1.34E-09 | B-cell_2 |
| SERPINF1.2 | 8.96E-14 | -0.94779834 | 0.029 | 0.509 | 1.57E-09 | B-cell_2 |
| OSBPL10 | 9.34E-14 | -0.86693343 | 0.397 | 0.804 | 1.64E-09 | B-cell_2 |
| IGLV5-45 | 1.11E-13 | -0.76641189 | 0.015 | 0.497 | 1.95E-09 | B-cell_2 |
| ABCA6 | 2.19E-13 | -0.8389172 | 0 | 0.47 | 3.84E-09 | B-cell_2 |
| SH3BP5.2 | 2.33E-13 | -0.83536707 | 0.5 | 0.857 | 4.09E-09 | B-cell_2 |
| MYCBP2 | 2.59E-13 | 0.770467448 | 0.809 | 0.457 | 4.55E-09 | B-cell_2 |
| APPL1.2 | 2.64E-13 | -0.82999041 | 0.353 | 0.765 | 4.63E-09 | B-cell_2 |
| SLC25A42 | 3.88E-13 | 0.383673163 | 0.353 | 0.093 | 6.82E-09 | B-cell_2 |
| WNT3.2 | 5.50E-13 | -0.94475629 | 0.015 | 0.476 | 9.65E-09 | B-cell_2 |
| RPS29 | 8.23E-13 | 0.463145341 | 1 | 1 | 1.45E-08 | B-cell_2 |
| VASP | 9.73E-13 | 0.641432141 | 0.647 | 0.294 | 1.71E-08 | B-cell_2 |
| PRDM2 | 1.09E-12 | -0.81100076 | 0.603 | 0.911 | 1.92E-08 | B-cell_2 |
| ZNF804A.2 | 1.23E-12 | -0.97709555 | 0.044 | 0.495 | 2.17E-08 | B-cell_2 |
| MYO1E.2 | 1.46E-12 | -0.79091923 | 0.044 | 0.5 | 2.56E-08 | B-cell_2 |
| HLA-DPA1 | 2.04E-12 | 0.912993333 | 0.956 | 0.956 | 3.57E-08 | B-cell_2 |
| H3F3B | 2.14E-12 | -0.7134921 | 0.985 | 1 | 3.76E-08 | B-cell_2 |
| RPS18 | 2.44E-12 | 0.619272821 | 1 | 1 | 4.28E-08 | B-cell_2 |
| MARCKSL1 | 3.65E-12 | -0.86587235 | 0.25 | 0.66 | 6.42E-08 | B-cell_2 |
| RPL39.1 | 4.81E-12 | 0.649039395 | 1 | 1 | 8.45E-08 | B-cell_2 |
| ZYX | 4.93E-12 | 0.303165819 | 0.221 | 0.044 | 8.66E-08 | B-cell_2 |
| MAN1A1 | 5.39E-12 | 0.482676869 | 0.191 | 0.035 | 9.47E-08 | B-cell_2 |
| MEF2C | 6.92E-12 | 0.82262116 | 0.853 | 0.599 | 1.21E-07 | B-cell_2 |
| PRKCB | 7.07E-12 | -0.68924403 | 0.721 | 0.94 | 1.24E-07 | B-cell_2 |
| RPL37A | 7.70E-12 | 0.389266271 | 1 | 1 | 1.35E-07 | B-cell_2 |
| ITM2C | 8.73E-12 | 0.687762383 | 0.191 | 0.035 | 1.53E-07 | B-cell_2 |
| TRAPPC2L | 1.27E-11 | -0.74853895 | 0.176 | 0.594 | 2.23E-07 | B-cell_2 |
| SNX22.2 | 1.40E-11 | 0.456670118 | 0.382 | 0.117 | 2.46E-07 | B-cell_2 |
| LINC01480 | 1.54E-11 | -0.91786106 | 0.147 | 0.568 | 2.70E-07 | B-cell_2 |
| CD9 | 1.80E-11 | -0.77414458 | 0.029 | 0.451 | 3.15E-07 | B-cell_2 |
| SPOCK2 | 2.20E-11 | -0.71237368 | 0.103 | 0.539 | 3.86E-07 | B-cell_2 |
| CRIP2.2 | 2.49E-11 | -0.83760954 | 0.176 | 0.598 | 4.37E-07 | B-cell_2 |
| SEC62.1 | 2.59E-11 | -0.71474167 | 0.618 | 0.919 | 4.54E-07 | B-cell_2 |
| MYO1F | 2.69E-11 | 0.386895754 | 0.324 | 0.091 | 4.73E-07 | B-cell_2 |
| ARGLU1 | 3.88E-11 | 0.698738978 | 0.897 | 0.692 | 6.81E-07 | B-cell_2 |
| IKZF3 | 3.93E-11 | -0.69997319 | 0.75 | 0.94 | 6.90E-07 | B-cell_2 |
| BTG2.1 | 7.85E-11 | -0.8588637 | 0.647 | 0.884 | 1.38E-06 | B-cell_2 |
| GSTP1 | 9.37E-11 | -0.55019375 | 0.397 | 0.834 | 1.65E-06 | B-cell_2 |
| RERE | 9.42E-11 | -0.77045335 | 0.265 | 0.646 | 1.65E-06 | B-cell_2 |
| DEF8 | 9.79E-11 | 0.261160734 | 0.191 | 0.037 | 1.72E-06 | B-cell_2 |
| CCDC191.1 | 1.09E-10 | -0.81062038 | 0.118 | 0.516 | 1.92E-06 | B-cell_2 |
| RPL5 | 1.12E-10 | 0.597831262 | 1 | 0.998 | 1.96E-06 | B-cell_2 |
| LINC01781 | 1.17E-10 | -0.87460542 | 0.147 | 0.547 | 2.05E-06 | B-cell_2 |
| MAST4 | 1.29E-10 | -0.66975943 | 0.044 | 0.442 | 2.27E-06 | B-cell_2 |
| HLA-DQB1 | 1.34E-10 | 0.980523773 | 0.882 | 0.855 | 2.36E-06 | B-cell_2 |
| BTN2A2 | 1.57E-10 | 0.317914056 | 0.279 | 0.073 | 2.76E-06 | B-cell_2 |
| COBLL1.1 | 1.58E-10 | -0.67686755 | 0.485 | 0.859 | 2.78E-06 | B-cell_2 |
| CCDC151.2 | 1.68E-10 | -0.7365684 | 0.015 | 0.415 | 2.95E-06 | B-cell_2 |
| IGLC2 | 1.77E-10 | 5.136453242 | 0.294 | 0.089 | 3.11E-06 | B-cell_2 |
| RPL31 | 1.88E-10 | 0.52151909 | 0.985 | 0.996 | 3.31E-06 | B-cell_2 |
| VIM.1 | 2.25E-10 | -0.61213757 | 0.882 | 0.988 | 3.95E-06 | B-cell_2 |
| TUBA1B | 2.34E-10 | -0.47919919 | 0.412 | 0.79 | 4.11E-06 | B-cell_2 |
| EPCAM.2 | 2.40E-10 | -0.68222209 | 0.029 | 0.419 | 4.22E-06 | B-cell_2 |
| CIRBP.2 | 2.43E-10 | -0.58450129 | 0.779 | 0.953 | 4.27E-06 | B-cell_2 |
| PLEKHA2.2 | 2.47E-10 | -0.7370018 | 0.324 | 0.685 | 4.34E-06 | B-cell_2 |
| TBC1D1 | 2.49E-10 | -0.70139753 | 0.279 | 0.663 | 4.38E-06 | B-cell_2 |
| MGAT5 | 2.73E-10 | -0.65077414 | 0.015 | 0.401 | 4.79E-06 | B-cell_2 |
| RPS12 | 3.02E-10 | 0.583512581 | 1 | 1 | 5.31E-06 | B-cell_2 |
| AC103591.3.2 | 3.15E-10 | 1.233170045 | 0.471 | 0.193 | 5.52E-06 | B-cell_2 |
| RPS17.1 | 3.26E-10 | 0.538076188 | 1 | 0.997 | 5.73E-06 | B-cell_2 |
| SEPTIN7 | 4.12E-10 | -0.63621985 | 0.809 | 0.96 | 7.23E-06 | B-cell_2 |
| TCL1A.2 | 4.17E-10 | -0.84962254 | 0.029 | 0.413 | 7.32E-06 | B-cell_2 |
| PDLIM1 | 4.88E-10 | -0.68331285 | 0.368 | 0.691 | 8.57E-06 | B-cell_2 |
| C4orf48 | 5.16E-10 | 0.43129354 | 0.426 | 0.156 | 9.07E-06 | B-cell_2 |
| TSTD1 | 5.48E-10 | -0.65508114 | 0.485 | 0.814 | 9.63E-06 | B-cell_2 |
| ORAI2 | 5.54E-10 | -0.7138575 | 0.368 | 0.738 | 9.72E-06 | B-cell_2 |
| HLA-DRB1.1 | 5.56E-10 | 1.032425796 | 0.956 | 0.977 | 9.77E-06 | B-cell_2 |
| HMGB1.2 | 5.67E-10 | 0.594390132 | 1 | 0.92 | 9.96E-06 | B-cell_2 |
| ZNF92 | 5.71E-10 | -0.71533592 | 0.132 | 0.52 | 1.00E-05 | B-cell_2 |
| APOBEC3H.2 | 6.36E-10 | -0.77381722 | 0.015 | 0.392 | 1.12E-05 | B-cell_2 |
| AMN.2 | 6.55E-10 | -0.65993683 | 0.029 | 0.407 | 1.15E-05 | B-cell_2 |
| SIK3 | 7.27E-10 | -0.64142736 | 0.176 | 0.552 | 1.28E-05 | B-cell_2 |
| GADD45B.2 | 8.34E-10 | -0.86179671 | 0.235 | 0.613 | 1.47E-05 | B-cell_2 |
| S100A6 | 9.47E-10 | -0.57831127 | 0.926 | 0.985 | 1.66E-05 | B-cell_2 |
| LCP1 | 9.49E-10 | 0.713610306 | 0.691 | 0.413 | 1.67E-05 | B-cell_2 |
| LY86 | 1.00E-09 | 0.393050538 | 0.338 | 0.109 | 1.76E-05 | B-cell_2 |
| PDCD4 | 1.02E-09 | -0.69813753 | 0.559 | 0.849 | 1.79E-05 | B-cell_2 |
| NIBAN3.2 | 1.02E-09 | -0.65424022 | 0.382 | 0.79 | 1.80E-05 | B-cell_2 |
| CD68 | 1.06E-09 | 0.257788722 | 0.162 | 0.031 | 1.85E-05 | B-cell_2 |
| NXPH4 | 1.27E-09 | -0.65990022 | 0.103 | 0.479 | 2.23E-05 | B-cell_2 |
| LIMS1 | 1.37E-09 | -0.73140286 | 0.191 | 0.583 | 2.40E-05 | B-cell_2 |
| CD86 | 1.81E-09 | 0.38793036 | 0.235 | 0.06 | 3.18E-05 | B-cell_2 |
| KLF6 | 1.94E-09 | -0.78231478 | 0.765 | 0.943 | 3.41E-05 | B-cell_2 |
| BCL7A | 2.14E-09 | -0.68179109 | 0.265 | 0.658 | 3.75E-05 | B-cell_2 |
| RRBP1.2 | 2.16E-09 | -0.77255515 | 0.235 | 0.638 | 3.79E-05 | B-cell_2 |
| C16orf74.2 | 2.72E-09 | -0.62803996 | 0.074 | 0.442 | 4.77E-05 | B-cell_2 |
| RIC3 | 2.96E-09 | -0.59535112 | 0.103 | 0.464 | 5.19E-05 | B-cell_2 |
| GAPT.2 | 3.09E-09 | 0.503472219 | 0.456 | 0.185 | 5.43E-05 | B-cell_2 |
| COCH | 3.35E-09 | 0.295643364 | 0.176 | 0.038 | 5.87E-05 | B-cell_2 |
| PTMA | 3.75E-09 | -0.38245318 | 1 | 1 | 6.59E-05 | B-cell_2 |
| ITGB1.2 | 3.97E-09 | 0.931606234 | 0.456 | 0.194 | 6.98E-05 | B-cell_2 |
| RAB11FIP1 | 4.93E-09 | -0.73777944 | 0.324 | 0.65 | 8.66E-05 | B-cell_2 |
| RPL34 | 5.68E-09 | 0.435691311 | 1 | 1 | 9.97E-05 | B-cell_2 |
| RPL36A | 7.94E-09 | 0.523182506 | 1 | 0.998 | 0.0001394 | B-cell_2 |
| UCP2.2 | 8.38E-09 | -0.61401378 | 0.897 | 0.957 | 0.0001472 | B-cell_2 |
| IFI16 | 8.74E-09 | -0.6442998 | 0.559 | 0.816 | 0.0001535 | B-cell_2 |
| ZDHHC21 | 9.23E-09 | 0.428804992 | 0.426 | 0.173 | 0.0001621 | B-cell_2 |
| NCR3.2 | 9.98E-09 | -0.60593662 | 0.147 | 0.497 | 0.0001753 | B-cell_2 |
| PTPRG | 1.08E-08 | -0.56794543 | 0 | 0.334 | 0.0001901 | B-cell_2 |
| HLA-DRA | 1.08E-08 | 0.660643314 | 0.926 | 0.995 | 0.0001902 | B-cell_2 |
| AP3B1 | 1.14E-08 | -0.67080588 | 0.294 | 0.63 | 0.0002008 | B-cell_2 |
| CDK14 | 1.22E-08 | -0.63409009 | 0.25 | 0.602 | 0.0002136 | B-cell_2 |
| RPL23 | 1.40E-08 | 0.382051276 | 1 | 1 | 0.0002458 | B-cell_2 |
| PAX5.2 | 1.45E-08 | -0.63107657 | 0.485 | 0.785 | 0.0002551 | B-cell_2 |
| PTPRJ | 1.47E-08 | -0.57561169 | 0.132 | 0.481 | 0.0002576 | B-cell_2 |
| PCGF5 | 1.53E-08 | -0.61471644 | 0.176 | 0.533 | 0.000269 | B-cell_2 |
| SMARCB1 | 1.58E-08 | -0.55395448 | 0.324 | 0.697 | 0.000277 | B-cell_2 |
| UBE2J1 | 1.59E-08 | 0.694946841 | 0.632 | 0.334 | 0.0002799 | B-cell_2 |
| SYNE2.1 | 1.66E-08 | -0.84342358 | 0.588 | 0.811 | 0.0002912 | B-cell_2 |
| FAM177B | 1.91E-08 | -0.52844937 | 0.015 | 0.344 | 0.0003357 | B-cell_2 |
| ARRB2 | 2.01E-08 | 0.321334987 | 0.235 | 0.065 | 0.0003525 | B-cell_2 |
| CD24.2 | 2.10E-08 | -0.63220217 | 0.25 | 0.611 | 0.0003681 | B-cell_2 |
| DENND3 | 2.53E-08 | 0.342848293 | 0.221 | 0.059 | 0.0004435 | B-cell_2 |
| LCK | 2.58E-08 | -0.53454609 | 0.059 | 0.405 | 0.0004522 | B-cell_2 |
| CCDC88A.1 | 2.59E-08 | -0.74662657 | 0.176 | 0.533 | 0.0004543 | B-cell_2 |
| ETV6 | 2.92E-08 | -0.57761945 | 0.132 | 0.47 | 0.0005131 | B-cell_2 |
| SMIM14.2 | 2.94E-08 | -0.74559875 | 0.515 | 0.789 | 0.0005163 | B-cell_2 |
| SINHCAF | 3.14E-08 | -0.60275695 | 0.265 | 0.597 | 0.0005522 | B-cell_2 |
| CCR7 | 3.53E-08 | -0.55757724 | 0.074 | 0.421 | 0.0006204 | B-cell_2 |
| SOD1.2 | 3.91E-08 | -0.57744842 | 0.691 | 0.882 | 0.0006862 | B-cell_2 |
| HLA-DQA1 | 3.94E-08 | 0.862135863 | 0.897 | 0.825 | 0.000692 | B-cell_2 |
| SLAMF7 | 3.96E-08 | 0.336831031 | 0.132 | 0.025 | 0.0006952 | B-cell_2 |
| RRAS2 | 4.20E-08 | -0.56232457 | 0.265 | 0.606 | 0.0007374 | B-cell_2 |
| SMPD3.2 | 4.74E-08 | -0.55996101 | 0.029 | 0.35 | 0.0008318 | B-cell_2 |
| CORO1B.2 | 4.76E-08 | -0.62016734 | 0.25 | 0.582 | 0.0008366 | B-cell_2 |
| BCL11A.2 | 4.84E-08 | -0.5508746 | 0.515 | 0.825 | 0.00085 | B-cell_2 |
| TXNIP.2 | 4.96E-08 | -0.60746145 | 0.941 | 0.985 | 0.0008701 | B-cell_2 |
| GPR183 | 4.97E-08 | 0.359994099 | 0.338 | 0.122 | 0.0008722 | B-cell_2 |
| AC245014.3.2 | 5.15E-08 | 1.102254986 | 0.456 | 0.223 | 0.0009042 | B-cell_2 |
| FCMR.2 | 7.06E-08 | -0.51398848 | 0.603 | 0.898 | 0.0012404 | B-cell_2 |
| RPS27A.2 | 7.27E-08 | -0.36618227 | 1 | 1 | 0.0012758 | B-cell_2 |
| ANP32B.2 | 7.50E-08 | 0.550420119 | 0.824 | 0.612 | 0.0013176 | B-cell_2 |
| SUB1 | 8.26E-08 | 1.223300721 | 0.765 | 0.623 | 0.0014503 | B-cell_2 |
| LGALS3.2 | 9.25E-08 | -0.58927488 | 0.074 | 0.395 | 0.0016235 | B-cell_2 |
| ARHGAP5 | 1.16E-07 | -0.58511335 | 0.191 | 0.521 | 0.0020432 | B-cell_2 |
| CXXC5 | 1.33E-07 | -0.43057149 | 0.426 | 0.775 | 0.0023323 | B-cell_2 |
| SERINC5 | 1.34E-07 | -0.51846975 | 0.044 | 0.352 | 0.0023548 | B-cell_2 |
| GLRX | 1.38E-07 | 0.426365054 | 0.412 | 0.178 | 0.002418 | B-cell_2 |
| NIN | 1.41E-07 | 0.479572755 | 0.515 | 0.254 | 0.0024834 | B-cell_2 |
| SRM | 2.18E-07 | 0.277190751 | 0.324 | 0.117 | 0.0038269 | B-cell_2 |
| RHBDL1 | 2.23E-07 | -0.54528381 | 0.059 | 0.362 | 0.0039109 | B-cell_2 |
| SARAF | 2.41E-07 | -0.5123086 | 0.426 | 0.73 | 0.0042344 | B-cell_2 |
| CD44 | 2.55E-07 | -0.53575922 | 0.456 | 0.748 | 0.004477 | B-cell_2 |
| JUN.2 | 2.61E-07 | -0.57680947 | 0.941 | 0.991 | 0.0045792 | B-cell_2 |
| TLN1.2 | 2.68E-07 | 0.549437036 | 0.765 | 0.477 | 0.004707 | B-cell_2 |
| NCL.1 | 2.85E-07 | -0.58518867 | 0.824 | 0.941 | 0.0050026 | B-cell_2 |
| AC044849.1.2 | 2.93E-07 | -0.62491221 | 0.206 | 0.534 | 0.005148 | B-cell_2 |
| CCDC144A | 3.02E-07 | 0.291736054 | 0.132 | 0.028 | 0.0053078 | B-cell_2 |
| PEG10.2 | 3.33E-07 | -0.66572497 | 0.103 | 0.409 | 0.0058532 | B-cell_2 |
| NFATC1 | 3.55E-07 | -0.4648026 | 0.059 | 0.364 | 0.0062329 | B-cell_2 |
| PNISR | 3.58E-07 | 0.479097381 | 0.941 | 0.83 | 0.0062847 | B-cell_2 |
| EEF1A1.2 | 3.69E-07 | 0.384843828 | 1 | 1 | 0.0064735 | B-cell_2 |
| RPS15A | 3.98E-07 | 0.35896563 | 1 | 1 | 0.0069964 | B-cell_2 |
| ITGB2-AS1 | 4.01E-07 | 0.353159739 | 0.279 | 0.099 | 0.0070434 | B-cell_2 |
| LHFPL2 | 4.17E-07 | -0.49170018 | 0.029 | 0.318 | 0.007316 | B-cell_2 |
| N4BP2L2.2 | 4.24E-07 | 0.56893871 | 0.809 | 0.668 | 0.0074393 | B-cell_2 |
| CD48 | 4.68E-07 | 0.605489598 | 0.809 | 0.666 | 0.0082198 | B-cell_2 |
| RHOA | 4.83E-07 | -0.47768228 | 0.824 | 0.94 | 0.008477 | B-cell_2 |
| CNR2 | 5.02E-07 | -0.51402677 | 0.147 | 0.454 | 0.0088124 | B-cell_2 |
| SKP1 | 5.48E-07 | -0.46759507 | 0.676 | 0.863 | 0.0096265 | B-cell_2 |
| COX7A2.2 | 6.28E-07 | 0.457291864 | 0.75 | 0.546 | 0.0110296 | B-cell_2 |
| DUSP22 | 6.43E-07 | -0.50836688 | 0.162 | 0.464 | 0.011291 | B-cell_2 |
| SNX10 | 6.52E-07 | -0.51793712 | 0.265 | 0.589 | 0.0114489 | B-cell_2 |
| MICOS13 | 6.58E-07 | -0.53401015 | 0.294 | 0.583 | 0.0115518 | B-cell_2 |
| SERPINB9P1 | 6.58E-07 | -0.47327012 | 0.029 | 0.311 | 0.0115591 | B-cell_2 |
| SP100.2 | 7.46E-07 | -0.4766386 | 0.721 | 0.927 | 0.0130935 | B-cell_2 |
| RPL14.2 | 7.74E-07 | -0.33209063 | 1 | 1 | 0.013584 | B-cell_2 |
| DUT | 7.85E-07 | -0.46389014 | 0.309 | 0.625 | 0.013783 | B-cell_2 |
| VOPP1 | 9.73E-07 | -0.5304799 | 0.338 | 0.633 | 0.017079 | B-cell_2 |
| GTF3C6 | 1.01E-06 | 0.293464454 | 0.338 | 0.136 | 0.0178085 | B-cell_2 |
| AC007384.1 | 1.13E-06 | -0.48212632 | 0.088 | 0.389 | 0.0197761 | B-cell_2 |
| CARMIL1 | 1.14E-06 | -0.42831408 | 0.015 | 0.283 | 0.0200527 | B-cell_2 |
| PMAIP1 | 1.16E-06 | -0.51497904 | 0.338 | 0.682 | 0.0203942 | B-cell_2 |
| AGPAT5 | 1.16E-06 | -0.52336049 | 0.132 | 0.42 | 0.0204105 | B-cell_2 |
| APP | 1.17E-06 | -0.42048042 | 0.015 | 0.283 | 0.0205424 | B-cell_2 |
| HINT1 | 1.18E-06 | -0.41010827 | 0.926 | 0.974 | 0.0207215 | B-cell_2 |
| GNG7.2 | 1.28E-06 | 0.360407369 | 0.544 | 0.279 | 0.0224966 | B-cell_2 |
| RPL27 | 1.35E-06 | 0.394350689 | 1 | 1 | 0.0237635 | B-cell_2 |
| CD37 | 1.38E-06 | -0.45470391 | 0.926 | 0.998 | 0.0242013 | B-cell_2 |
| RHOB.2 | 1.39E-06 | -0.54255667 | 0.368 | 0.686 | 0.0244802 | B-cell_2 |
| IFNGR1 | 1.40E-06 | 0.281140693 | 0.176 | 0.049 | 0.0245163 | B-cell_2 |
| PHF14 | 1.41E-06 | -0.49238901 | 0.294 | 0.563 | 0.024769 | B-cell_2 |
| GNG11 | 1.41E-06 | -0.45528209 | 0 | 0.259 | 0.0248401 | B-cell_2 |
| RAPGEF1.2 | 1.54E-06 | 0.403340053 | 0.5 | 0.253 | 0.0270721 | B-cell_2 |
| CDC42SE2 | 1.70E-06 | -0.46992883 | 0.353 | 0.672 | 0.0298065 | B-cell_2 |
| TIMM8B | 1.75E-06 | 0.330238481 | 0.235 | 0.08 | 0.0307028 | B-cell_2 |
| USP34 | 1.84E-06 | -0.47994844 | 0.397 | 0.65 | 0.0322559 | B-cell_2 |
| RPS24.2 | 1.85E-06 | -0.27268499 | 1 | 1 | 0.0325421 | B-cell_2 |
| SPN | 1.87E-06 | 0.434255036 | 0.147 | 0.037 | 0.0328937 | B-cell_2 |
| MS4A1.2 | 1.89E-06 | 0.819404229 | 0.838 | 0.981 | 0.0331729 | B-cell_2 |
| SQSTM1.2 | 1.90E-06 | -0.51077962 | 0.338 | 0.631 | 0.0333224 | B-cell_2 |
| CENPM.2 | 1.91E-06 | -0.49378106 | 0.088 | 0.363 | 0.0335881 | B-cell_2 |
| EIF5 | 1.99E-06 | -0.5318584 | 0.544 | 0.767 | 0.0349264 | B-cell_2 |
| YBX3 | 2.14E-06 | -0.51872336 | 0.25 | 0.556 | 0.0376405 | B-cell_2 |
| TMBIM6.1 | 2.26E-06 | -0.39680854 | 0.676 | 0.856 | 0.0396812 | B-cell_2 |
| GRN | 2.28E-06 | 0.514048774 | 0.412 | 0.194 | 0.0400159 | B-cell_2 |
| ANXA11 | 2.28E-06 | -0.47025098 | 0.368 | 0.644 | 0.0400263 | B-cell_2 |
| SPINT2 | 2.32E-06 | -0.43549626 | 0.324 | 0.641 | 0.0406882 | B-cell_2 |
| SRSF11 | 2.35E-06 | 0.560067521 | 0.691 | 0.498 | 0.0412477 | B-cell_2 |
| CCDC85B | 2.41E-06 | 0.343088642 | 0.515 | 0.27 | 0.0422963 | B-cell_2 |
| AKAP13.2 | 2.46E-06 | -0.50743703 | 0.735 | 0.905 | 0.0432279 | B-cell_2 |
| NCF1 | 2.47E-06 | -0.46162039 | 0.676 | 0.87 | 0.0433645 | B-cell_2 |
| SH3YL1 | 2.58E-06 | -0.47786217 | 0.176 | 0.465 | 0.0453617 | B-cell_2 |
| GGA1 | 2.61E-06 | -0.48097431 | 0.235 | 0.541 | 0.0457664 | B-cell_2 |
| AC246817.1 | 2.61E-06 | -0.42143222 | 0.015 | 0.27 | 0.0458074 | B-cell_2 |
| GNPTAB | 2.63E-06 | -0.50433622 | 0.176 | 0.456 | 0.0461034 | B-cell_2 |
| MRNIP | 2.63E-06 | -0.49059726 | 0.147 | 0.424 | 0.0461307 | B-cell_2 |
| ADTRP.1 | 2.67E-06 | -0.42068343 | 0 | 0.249 | 0.0468856 | B-cell_2 |
| EIF4G2 | 2.68E-06 | -0.46497818 | 0.603 | 0.84 | 0.0470667 | B-cell_2 |
| POMC | 2.72E-06 | -0.44472061 | 0.103 | 0.381 | 0.0478167 | B-cell_2 |

**Supplemental References**

1. Hao Y, Hao S, Andersen-Nissen E, Mauck WM, 3rd, Zheng S, Butler A*, et al.* Integrated analysis of multimodal single-cell data. *Cell* 2021 Jun 24; **184**(13)**:** 3573-3587 e3529.

2. McGinnis CS, Murrow LM, Gartner ZJ. DoubletFinder: Doublet Detection in Single-Cell RNA Sequencing Data Using Artificial Nearest Neighbors. *Cell Syst* 2019 Apr 24; **8**(4)**:** 329-337 e324.

3. Becht E, McInnes L, Healy J, Dutertre CA, Kwok IWH, Ng LG*, et al.* Dimensionality reduction for visualizing single-cell data using UMAP. *Nat Biotechnol* 2018 Dec 3.

4. Tickle T, Tirosh I, Georgescu C, Brown M, Haas B. inferCNV of the Trinity CTAT Project. *Klarman Cell Observatory, Broad Institute of MIT and Harvard* 2019.

5. Liberzon A, Birger C, Thorvaldsdottir H, Ghandi M, Mesirov JP, Tamayo P. The Molecular Signatures Database (MSigDB) hallmark gene set collection. *Cell Syst* 2015 Dec 23; **1**(6)**:** 417-425.
