# Supplementary figures and images for: Pevonedistat, a Nedd8-activating enzyme inhibitor, in combination with ibrutinib in patients with relapsed/refractory B-cell non-Hodgkin lymphoma
Source: Blood Cancer J. 2023 Jan 11;13(1):9. doi: 10.1038/s41408-022-00763-w (PMC9834208; doi:10.1038/s41408-022-00763-w)

## Slide 1
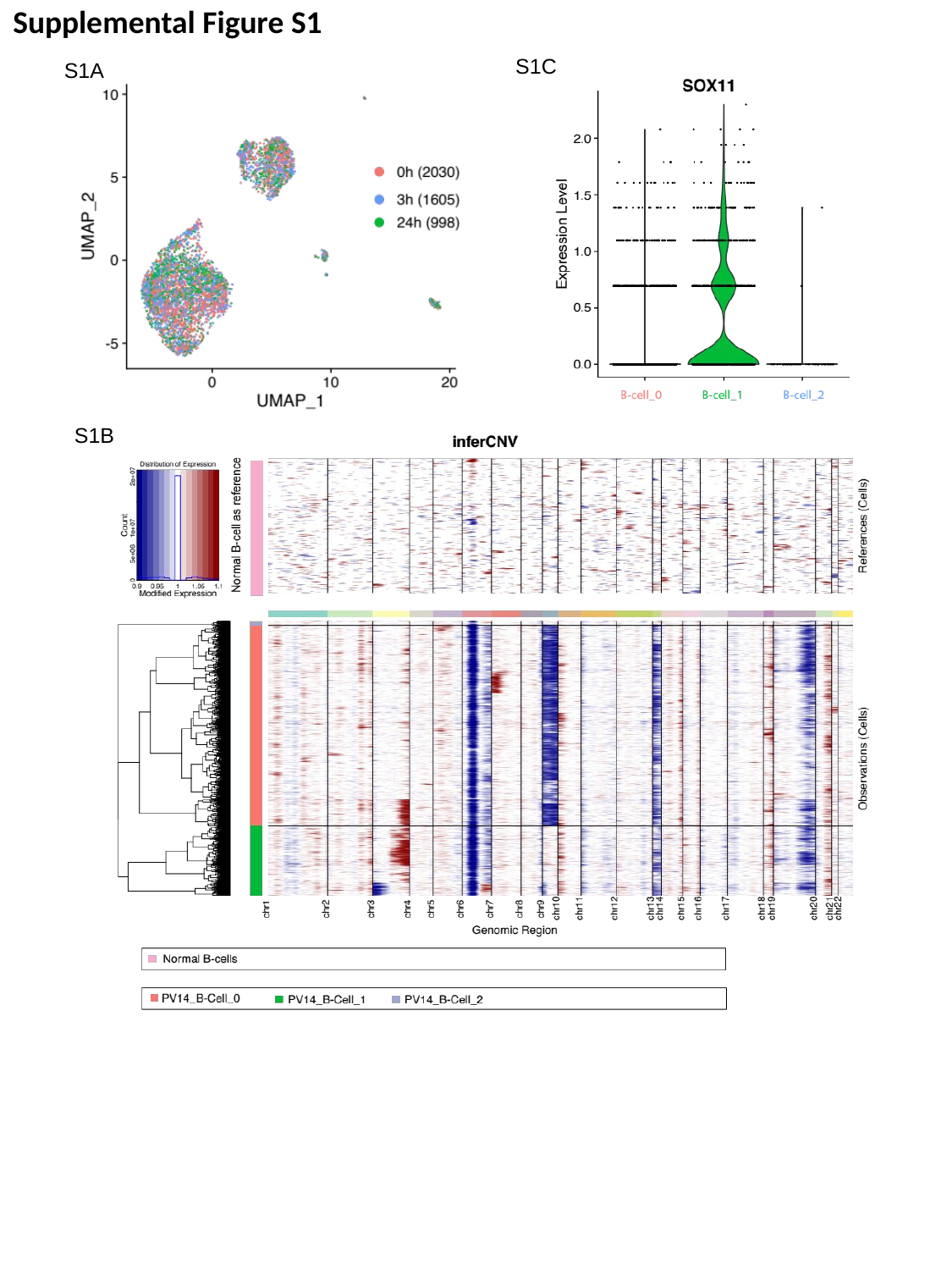

Supplemental Figure S1
S1C
S1A
S1B

## Slide 2
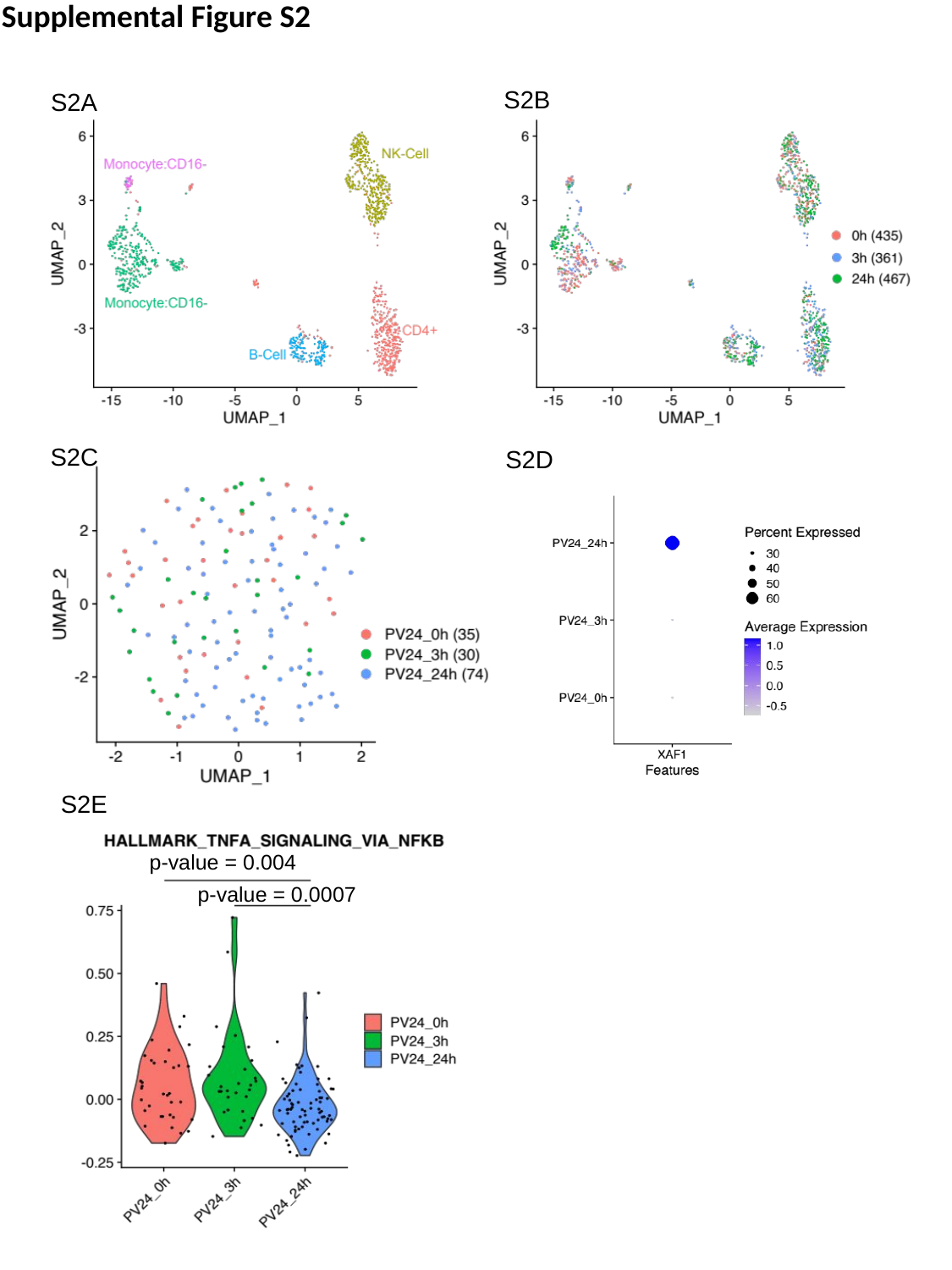

Supplemental Figure S2
S2B
S2A
S2C
S2D
S2E
p-value = 0.004
p-value = 0.0007

Supplement: Supplementary file 2 — Supplementary Figures 1-2 [file 41408_2022_763_MOESM2_ESM.pptx]
